# AxialTAD: an axial contrast architecture for TAD boundary prediction from Hi-C

**DOI:** 10.1093/bib/bbag603

**Published:** 2026-11-02

**Authors:** Woo-Young Choi, Je-Keun Rhee

**Affiliations:** Department of Bioinformatics & Life Science, Soongsil University, 369, Sangdo-ro, Dongjak-gu, Seoul 06978, Republic of Korea; Department of Bioinformatics & Life Science, Soongsil University, 369, Sangdo-ro, Dongjak-gu, Seoul 06978, Republic of Korea; AI-Bio Convergence Research Institute, Soongsil University, 369, Sangdo-ro, Dongjak-gu, Seoul 06978, Republic of Korea

**Keywords:** Hi-C, topologically associating domains, TAD boundary prediction, deep learning, inductive bias, chromatin organization

## Abstract

Topologically associating domains (TADs) are fundamental units of 3D chromatin organization, and accurate identification of TAD boundaries from Hi-C contact matrices remains an active challenge. Recent deep learning approaches have advanced substantially, but these rely on increasingly large architectures, raising the question of how much of this capacity boundary-centric analysis requires. Here we propose AxialTAD, a compact deep learning model centered on an Axial Contrast Module (ACM) that explicitly encodes directional contrast in contact intensity along the row and column axes of local Hi-C patches, an operator aligned with the geometric signature of TAD boundaries. Evaluated on four human cell lines (GM12878, HeLa-S3, IMR-90, K562) within cell lines and under leave-one-cell-out generalization, AxialTAD performed comparably with a recent convolutional neural network (CNN)+Transformer baseline (deepTAD) while using 76%–80% fewer trainable parameters (113K–137K versus 571K). A parameter-matched conventional CNN achieved similar F1 to AxialTAD in the human 25 kb experiments. When trained and evaluated within each of two mouse datasets, AxialTAD outperformed both this control and deepTAD. Orthogonal validation against CTCF and cohesin ChIP-seq supported the biological plausibility of the predicted boundaries. These results indicate that, within the range of architectures examined, a large architecture is not necessary to reach this level of boundary prediction.

## Introduction

High-throughput chromosome conformation capture (Hi-C) enables genome-wide measurement of 3D chromatin organization by quantifying pairwise contact frequencies between genomic loci [1]. Hi-C contact maps reveal multiple layers of chromatin structure, including megabase-scale A/B compartments [2], sub-megabase topologically associating domains (TADs) [3], and kilobase-resolution chromatin loops [4, 5].

In particular, TADs appear as diagonal blocks of enriched interaction frequencies within Hi-C contact matrices. Within these domains, chromatin interactions occur frequently, whereas interactions across domain boundaries are insulated. TADs serve as fundamental regulatory units, confining enhancer–promoter interactions and facilitating co-regulation of genes, while their disruption is often associated with developmental disorders and cancer [6, 7]. Therefore, accurate identification of TAD boundaries is a prerequisite for interpreting the functional implications of 3D genome architecture.

Despite the biological significance of TADs, identifying their boundaries remains a computational challenge. A range of conventional methods, including the Directionality Index (DI) [3], Insulation Score (IS) [8], TopDom [9], HiCExplorer [10, 11], and OnTAD [12], have been developed to detect boundaries based on local changes in contact frequency. However, these methods typically rely on hand-crafted statistical features and linear scanning windows. Consequently, predictions vary significantly depending on the choice of algorithm and parameters (e.g. window size), leading to low reproducibility across different studies [13, 14].

Recently, deep learning approaches have been introduced to TAD boundary prediction to learn discriminative features directly from Hi-C contact patterns, with data-driven models capturing complex nonlinear interactions that hand-crafted statistics struggle to represent [15]. A range of learning-based methods has since been developed, including base-pair-resolution boundary prediction from genomic annotations [16], region-based convolutional detection of domain boundaries [17], and attention-based domain identification [18]. Furthermore, sequence- and contact-based models have been developed for broader 3D genome tasks, including diffusion- and transformer-based interaction prediction [19] and large-scale Hi-C foundation models [20]. Among these, deepTAD [21] established a boundary-centric classification framework using a convolutional neural network (CNN)+Transformer architecture. This reduces TAD boundary detection to a tractable local-context classification task and is the framing adopted in the present study.

With the architecture established, an open question for boundary-centric Hi-C classification is what architectural inductive bias most efficiently captures boundary-relevant patterns from the small local context that this framing provides. TAD boundaries appear in Hi-C contact matrices as the corners of square-shaped diagonal blocks, where contact intensity transitions abruptly along two orthogonal axes—from high intra-domain contacts inside the block to low inter-domain contacts across the boundary [3, 8]. This directional, axis-aligned contrast is a defining geometric signature of TAD boundaries that recurs across cell types and Hi-C resolutions, and is the same signal that classical DI [3] and IS [8] methods quantify with hand-crafted statistics. While general-purpose CNN+Transformer architectures can in principle rediscover this signal from data, doing so allocates model capacity to a feature that can be specified *a priori* from the geometry of the problem. Encoding axis-aligned contrast as an explicit, learnable inductive bias—operators that compute directional differences along each cardinal axis and assess their spatial coherence—offers an alternative design point: model capacity is reserved for variations around this geometric template, rather than spent on rediscovering the template itself.

In this study, we propose AxialTAD, a compact deep learning model that builds on the boundary-centric framing and replaces its general-purpose CNN+Transformer core with an Axial Contrast Module (ACM), an explicit, learnable encoding of axis-aligned contrast around candidate boundary bins. We evaluate AxialTAD on four human cell lines under three reference boundary definitions in within-cell-line and leave-one-cell-out (LOCO) settings, and additionally train and evaluate the models within two mouse datasets, with CTCF and cohesin enrichment providing complementary biological evidence. AxialTAD achieves boundary prediction performance comparable with deepTAD within human cell lines and under LOCO generalization, while using 76%–80% fewer trainable parameters. Within the two mouse datasets, AxialTAD achieved higher F1 than both a parameter-matched convolution and the larger deepTAD. Component ablation indicates that the ACM contributes at the block level, since removing the module as a whole produces a significant F1 drop while ablating individual sub-components remains within seed-level variance. These results support explicit axis-aligned contrast modeling as a compact design point for boundary-centric Hi-C classification.

## Methods

### Hi-C data acquisition and preprocessing

Hi-C contact matrices for four human cell lines were obtained from the 4D Nucleome (4DN) Data Portal: GM12878 (4DNFI1UEG1HD), HeLa-S3 (4DNFICEGAHRC), IMR-90 (4DNFIH7TH4MF), and K562 (4DNFITUOMFUQ) [22]. Contact matrices for chromosomes 1–22 were extracted at 25 kb resolution with Knight–Ruiz (KR) balancing applied [23]. Five chromosomes for which KR balancing failed at 25 kb resolution (GM12878 chr7, GM12878 chr10, IMR-90 chr18, K562 chr7, and K562 chr9) were excluded from analysis, yielding 83 chromosome-level matrices in total.

Because no universally accepted gold-standard annotation of TAD boundaries is currently available, three reference boundary sets were constructed to evaluate model performance under different labeling criteria. The first reference set (filtered) retained only OnTAD boundaries at TAD level 1 with TAD score *≥ * 2. The second reference set (unfiltered) retained all OnTAD boundaries at TAD level *≥ * 1, excluding only OnTAD’s level 0 internal annotations. The third reference set (intersection) was constructed as the intersection of unfiltered OnTAD boundaries with TAD boundaries called by HiCExplorer v3.7.6 [10] using hicFindTADs with default 25 kb parameters (--minDepth 75000 --maxDepth 250000 --step 25000 --correctForMultipleTesting fdr --thresholdComparisons 0.01). OnTAD v1.4 [12] was run with its default parameters (-maxsz 200 -minsz 3 -penalty 0.1 -ldiff 1.96 -lsize 5); the -hic_norm flag was omitted as it applies only to .hic-input mode in OnTAD v1.4, with the default NONE normalization appropriate for the already KR-balanced text matrices used as input. Models were trained and evaluated separately under each reference set.

For the evaluation on mouse datasets, mouse embryonic stem cell (mESC) Micro-C [24] and CH12-LX *in situ* Hi-C [4] were processed identically to the human data (at 25 kb resolution using KR balancing and the intersection reference). Each dataset was evaluated using leave-one-chromosome-out cross-validation over chromosomes 1–19. For test chromosome *k*, chromosome *(k \bmod 19)+1* was used for validation and the remaining 17 chromosomes for training.

### Sample generation and labeling

For each chromosome-level Hi-C contact matrix, candidate bins along the diagonal were enumerated, and a square submatrix of size 15 *× * 15 centered at each candidate bin was extracted as model input. The 15 *× * 15 size was chosen to provide sufficient local context around the candidate boundary while keeping the input compatible with lightweight architectures. Each candidate bin received a binary label under each of the three reference boundary sets; models were trained and evaluated separately under each reference.

Train, validation, and test sets were defined using a fixed chromosome-level split applied identically across all four cell lines. Chromosomes 20, 21, and 22 were held out as the test set, aligning with the test split adopted in the original deepTAD study [21]. Chromosomes 16, 17, and 19 were used as the validation set; chromosome 18 was excluded due to KR balancing failure in IMR-90. The remaining chromosomes 1 through 15, excluding the cell-line-specific KR-failed chromosomes, formed the training set, yielding 56 chromosome–cell-line pairs for training, 12 for validation, and 12 for testing. All models were trained for a fixed number of epochs with model selection based on best validation F1; the training and hyperparameter optimization protocols are described below.

To address the imbalance between boundary and non-boundary samples, positive samples in the training set were augmented by 90$^\circ $ clockwise rotation in a label-preserving manner (the boundary status of the central bin is invariant under rotation), doubling the positive count. Negative samples were then randomly subsampled to achieve a 4:1 negative-to-positive ratio in the final training set. Both the rotation augmentation and the 4:1 ratio follow the protocol of deepTAD [21]. Negative subsampling was controlled by an explicit random seed matched to the model initialization seed. No augmentation was applied to validation or test sets. Each *15 × 15* input patch was standardized row-by-row prior to model input, following the deepTAD preprocessing protocol [21].

The contribution of rotation augmentation was examined through a controlled ablation that separates the rotation effect from the training-data-size confound, using two no-rotation control conditions (Supplementary Note S1).

### AxialTAD architecture

AxialTAD is a compact deep learning model composed of three sequential components: a convolutional encoder that extracts local features from the input submatrix, the proposed ACM that explicitly encodes axis-aligned contrast as an inductive bias, and a classification head that produces a boundary probability. The model therefore predicts per-bin boundary labels rather than assembling complete or hierarchical TAD structures. An overview of the complete architecture is provided in Fig. 1.

**Figure 1.**
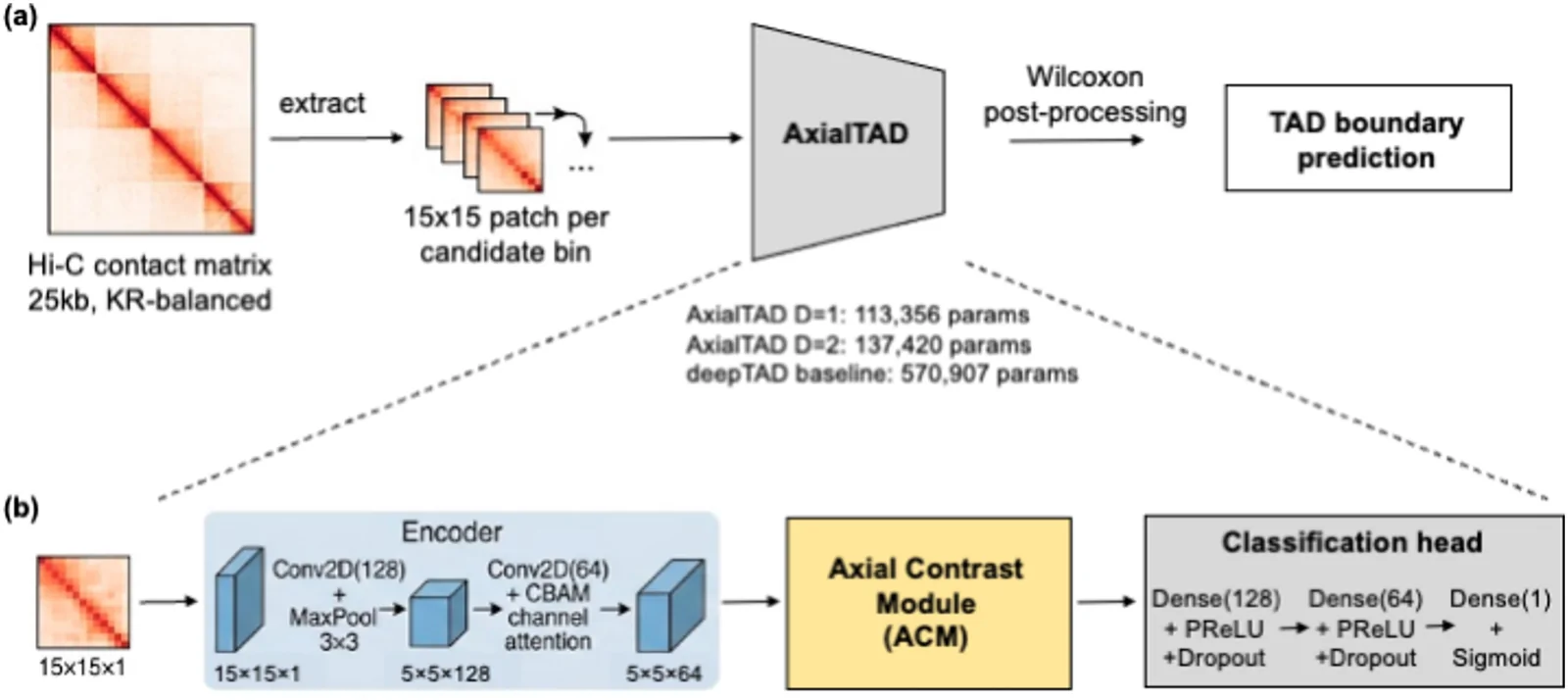
Overview of the AxialTAD architecture. (a) End-to-end pipeline: KR-balanced Hi-C contact matrices at 25 kb resolution are partitioned into *15 × 15* submatrices centered at each candidate boundary bin, processed by the AxialTAD model, and the resulting per-bin boundary probabilities are post-processed by a Wilcoxon rank-sum filter, yielding final TAD boundary positions. (b) AxialTAD network architecture: a convolutional encoder (Conv2D 128, *3 × 3* MaxPool, Conv2D 64 with CBAM channel attention) compresses the input to a $5 \times 5 \times 64$ feature map; the ACM—the novel component of AxialTAD—reduces this to a single center logit through axis-aligned contrast operations, and a three-layer classifier head (Dense 128, Dense 64, Dense 1 with PReLU activations, dropout 0.4, and sigmoid output) produces the final boundary probability.

The convolutional encoder takes a *15 × 15* submatrix as input and applies two convolutional layers separated by a single max-pooling step. The first convolutional layer (128 channels, *3 × 3* kernel, same padding, ReLU activation) is followed by max-pooling (*3 × 3* kernel, stride 3), reducing the spatial dimensions from *15 × 15* to *5 × 5*. The second convolutional layer (64 channels, *3 × 3* kernel, same padding, ReLU activation) produces a feature map of shape $5 \times 5 \times 64$. The Channel Attention Module of Convolutional Block Attention Module (CBAM) [25] is then applied to refine channel-wise feature representation, producing $X \in \mathbb{R}^{H \times W \times C}$ with *H = W = 5* and *C = 64*. The spatial attention component of CBAM is deliberately omitted: CBAM spatial attention produces an isotropic spatial weighting that does not distinguish row and column directions, whereas the subsequent ACM is designed to encode anisotropic axial contrast as an explicit inductive bias, and combining the two would partially obscure this directionality. The ACM then encodes directional changes in contact intensity through five sequential operations on *X*.

First, for each shift size $d \in \{1, \dots , D\}$, the input is shifted along each of the four cardinal directions (up, down, left, right) with zero-padding at the borders to preserve spatial dimensions:


(1) \begin{align*} & X^{down,d}[i, j] = \begin{cases} X[i-d, j] & \textrm{if}\ i - d \geq 0 \\ 0 & \textrm{otherwise} \end{cases} \end{align*}

(2) \begin{align*} & X^{up,d}[i, j] = \begin{cases} X[i+d, j] & \textrm{if}\ i + d < H \\ 0 & \textrm{otherwise} \end{cases} \end{align*}

with analogous definitions for $X^{left,d}$ and $X^{right,d}$. For each shifted tensor, both the signed difference $X^{s,d} - X$ and the absolute difference $|X^{s,d} - X|$ are computed, providing two complementary representations of axial contrast. The hyperparameter *D* controls the shift range: *D = 2* (default) uses shifts of 1 and 2 bins, while *D = 1* (alternative) uses only 1-bin shifts. The shift range is bounded by *D ≤ 2* for a *5 × 5* feature map.

Second, the difference tensors are concatenated along the channel dimension into two per-axis stacks:


(3) \begin{align*} F_{v} = \mathrm{Concat}_{c} \big(\{ & X^{down,d}-X,\ X^{up,d}-X, |X^{down,d}-X|,\ |X^{up,d}-X| \}_{d=1}^{D}\big) \end{align*}

(4) \begin{align*} F_{h} = \mathrm{Concat}_{c} \big(\{ & X^{right,d}-X,\ X^{left,d}-X, |X^{right,d}-X|,\ |X^{left,d}-X| \}_{d=1}^{D}\big) , \end{align*}

where $F_{v}, F_{h} \in \mathbb{R}^{H \times W \times 4DC}$. $F_{v}$ aggregates vertical-direction (row-axis) shifts and $F_{h}$ aggregates horizontal-direction (column-axis) shifts. Each stack has channel dimension *4DC* (*= 512* in the default configuration).

Third, each stack is summarized through a two-stage axis-specific convolution that evaluates whether contrast evidence is spatially coherent along the boundary axis. The vertical-difference stack $F_{v}$ is processed by a separable convolution with kernel $3 \times k_{row}$ ($k_{row} = 5$), followed by a *1 × 1* convolution with *M = 32* channels, both with ReLU activation and same-padding:


(5) \begin{align*} R = & \ \mathrm{ReLU}\big(\mathrm{Conv}_{1 \times 1, M}\big( \mathrm{ReLU}\big(\mathrm{SepConv}_{3 \times k_{row}}(F_{v})\big)\big)\big)\end{align*}

The kernel is wider along the column axis ($k_{row} = 5$) to assess horizontal continuity of vertical contrast, with a small extent along the row axis (3) to allow local smoothing. The horizontal-difference stack $F_{h}$ is processed analogously with a kernel $k_{col} \times 3$ ($k_{col} = 5$), producing $C \in \mathbb{R}^{H \times W \times M}$ that assesses vertical continuity of horizontal contrast. The two-stage design (separable convolution followed by *1 × 1* convolution) separates spatial filtering from channel mixing, keeping parameter count low while preserving capacity to compose features across the *4DC* input channels.

Fourth, a per-axis spatial gating mechanism modulates feature reliability. Two independent gates are computed from *R* and *C*, respectively, each producing a single-channel sigmoid map broadcast over channels:


(6) \begin{align*} G_{R} &= \sigma\left(\mathrm{Conv}_{1 \times 1, 1}(R)\right) \in [0, 1]^{H \times W \times 1} \nonumber \\ G_{C} &= \sigma\left(\mathrm{Conv}_{1 \times 1, 1}(C)\right) \in [0, 1]^{H \times W \times 1} \nonumber \\ \tilde{R} &= R \odot G_{R}, \quad \tilde{C} = C \odot G_{C}\end{align*}

The per-axis (rather than combined) gating allows the model to suppress noisy regions independently along each axis: a spatial position may have reliable vertical contrast evidence but unreliable horizontal evidence, or vice versa, and the two gates address these cases independently.

Fifth, the gated axis features are fused and reduced to a logit map. The concatenated tensor $\mathrm{Concat}_{c}(\tilde{R}, \tilde{C}) \in \mathbb{R}^{H \times W \times 2M}$ passes through a two-stage *1 × 1* convolution head (first layer: *M* channels with ReLU; second layer: 1 channel, linear), producing a logit map $L \in \mathbb{R}^{H \times W \times 1}$. The boundary score for the candidate bin is extracted as the logit value at the central coordinate, $s = L[\lfloor H/2 \rfloor , \lfloor W/2 \rfloor , 0]$. Center extraction is appropriate because the input submatrix is centered at the candidate bin and the binary boundary label is defined for that bin specifically; spatial aggregation alternatives (e.g. global average pooling) would mix the boundary-specific signal with non-boundary positions.

The boundary score *s* is processed by the classification head, which consists of three fully connected layers (128, 64, and 1 units) with PReLU activation and dropout (rate 0.4) on the first two layers and a sigmoid output on the third, producing the final boundary probability. With *D = 2*, the complete AxialTAD model contains 137 420 trainable parameters; the *D = 1* configuration contains 113 356 parameters, corresponding to a reduction of *∼ *76% and 80% relative to the deepTAD baseline (570 907 parameters at *15 × 15* input). A component-wise parameter breakdown is provided in Table 1.

**Table 1:** Architecture variants evaluated in this study, grouped by category

Variant	Category	Parameters	Key modification
AxialTAD (*D=1*)	AxialTAD primary	113 356	ACM with shift size *D=1*
AxialTAD (*D=2*)	AxialTAD primary	137 420	ACM with shift size *D=2* (default)
ACM-removed	AxialTAD ablation	87 050	ACM replaced with fusion-and-logit head only
$F_{v}$ -only	AxialTAD ablation	111 211	Only vertical-shift difference stack retained
$F_{h}$ -only	AxialTAD ablation	111 211	Only horizontal-shift difference stack retained
No-gating	AxialTAD ablation	137 354	Sigmoid gating maps $G_{R}, G_{C}$ removed
deepTAD (*15 × 15*)	deepTAD baseline	570 907	CNN + CBAM + single-token Transformer (MHA)
deepTAD (*10 × 10*)	deepTAD baseline	529 947	Same architecture, original patch size
Multi-token (4)	deepTAD-family	554 523	Feature map pooled to *2 × 2* (4 tokens)
Multi-token (25)	deepTAD-family	898 587	Feature map pooled to *5 × 5* (25 tokens)
Multi-token (100)	deepTAD-family	2127 387	Feature map pooled to *10 × 10* (100 tokens)
MHA-ablation	deepTAD-family	323 611	MHA *→ * Dense ($d_{\mathrm{model}}=128$)
plain CNN (*3× 3*)	parameter-matched control	137 658	ACM replaced with *3× 3* convolution
pointwise (*1× 1*)	parameter-matched control	137 408	ACM replaced with *1× 1* convolution

### Baseline and ablation variants

The deepTAD baseline architecture [21] was used as the direct comparison for AxialTAD. The implementation was taken verbatim from the public repository (https://github.com/xiaoyan-wang99/deepTAD); modifications were limited to parameterizing the input patch size to enable evaluation at both *15 × 15* (primary, matching AxialTAD’s input) and *10 × 10* (the original Wang *et al*. [21] configuration, evaluated as a supplementary patch-size sensitivity check). The architecture combines a convolutional feature extractor with a CBAM [25] followed by a single-token Transformer encoder with multi-head attention (MHA); the model and training procedure were preserved as released. The *15 × 15* deepTAD baseline contains 570 907 trainable parameters and the *10 × 10* baseline contains 529 947 parameters.

To assess which components of the ACM drive performance, four AxialTAD-internal ablation variants were constructed at the *15 × 15* patch size. All four variants share the encoder, fusion-and-logit head, center extraction, and classification head with the full AxialTAD architecture, differing only in the configuration of the four-step ACM. In the ACM-removed variant, the entire ACM (shift, stack, axis-specific convolution, and gating) is removed; the encoder output is passed directly through the fusion-and-logit head, retaining the two-stage *1 × 1* convolutional structure (*M*-channel ReLU followed by 1-channel linear) and center extraction. In the $F_{v}$-only variant, only the vertical-shift difference stack $F_{v}$ and its associated convolution are retained, while $F_{h}$ and the horizontal-axis branch are omitted; the $F_{h}$-only variant is the symmetric counterpart, retaining only $F_{h}$ and the horizontal-axis branch while omitting $F_{v}$ and the vertical-axis branch. In the no-gating variant, the sigmoid gating maps $G_{R}$ and $G_{C}$ are removed, and ungated *R* and *C* are passed directly to the fusion step.

To separate the contribution of the axial operators from that of overall model capacity, two parameter-matched control architectures were constructed at the *15 × 15* patch size. Both share the encoder, fusion-and-logit head, center extraction, and classification head with AxialTAD (*D=2*) and replace only the axial operators at the ACM position: the plain-CNN control substitutes a standard *3 × 3* convolution (a local spatial operation without the explicit axial form), and the pointwise control substitutes a *1 × 1* convolution (no spatial mixing). Both are matched to AxialTAD (*D=2*) in parameter count (*∼ *137K).

To contextualize AxialTAD against alternative architectures within the deepTAD family, four deepTAD-derived variants were additionally evaluated at the *15 × 15* patch size. The multi-token Transformer variants use max-pooling and average-pooling to reduce the convolutional feature map to spatial grids of *2 × 2*, *5 × 5*, or *10 × 10* (yielding 4, 25, or 100 tokens, respectively) prior to the Transformer encoder, enabling token-to-token attention computation across spatial positions. The MHA-ablation variant replaces the MHA layer in the deepTAD baseline with a Dense layer of dimension $d_{\mathrm{model}} = 128$, preserving the surrounding layer normalization, feed-forward, and residual structure.

All variants used the same input patch size, training protocol, and Wilcoxon post-processing as AxialTAD and the deepTAD baseline. These variants and their AxialTAD (D = 2) anchor were trained as a single batch, so that the reported differences are seed-wise paired comparisons within that batch. Parameter counts and modification summaries for all variants are provided in Table 1.

### Training and hyperparameter optimization

All models were trained using the Adam optimizer [26] with binary cross-entropy loss (label smoothing 0.01); convolutional and dense layers were initialized with the He uniform initializer [27]. Each training run used a fixed duration of 30 epochs without explicit early stopping, selected based on preliminary epoch-sensitivity experiments.

To prevent test-set contamination in model selection, a validation-driven training protocol was adopted. At the end of every epoch, binary F1 was computed on the held-out validation set (12 chromosome–cell-line pairs); whenever validation F1 improved over the prior best, the corresponding model weights were saved as the running checkpoint. Test-set inference used the best-validation-F1 epoch weights rather than the final-epoch weights. This protocol prevents test-set leakage in model selection, ensuring that no test-set information influences hyperparameter or epoch selection.

Hyperparameter optimization was performed over a grid of 16 (learning rate, batch size) combinations spanning learning rate $\in \{3 \times 10^{-5}, 1 \times 10^{-4}, 3 \times 10^{-4}, 1 \times 10^{-3}\}$ and batch size $\in \{64, 128, 256, 512, 1024\}$, with four corner combinations excluded (large learning rate paired with large batch size and small learning rate paired with small batch size, regions known to produce unstable training dynamics); the complete grid is provided in Supplementary Table S1. Each combination was trained with a single seed (seed = 123) on the intersection reference, and the configuration achieving the highest validation F1 was selected as the architecture-specific *tuned* hyperparameter setting (Supplementary Table S2). Two hyperparameter conditions were evaluated in the main analyses: a *default* setting (learning rate $3 \times 10^{-4}$, batch size 128, matching the original deepTAD configuration [21]) and the *tuned* setting identified per architecture by the grid search.

Predictions on the test set were post-processed using a Wilcoxon rank-sum filter following the protocol of Wang *et al*. [21]. For each predicted boundary, gap regions (centromeric and heterochromatic bins with zero 17-bin row-sum windows) were first flagged and excluded. The contact matrix was then standardized per anti-diagonal, and for each non-gap candidate bin a Wilcoxon rank-sum test was computed comparing the contact-intensity distribution of the diamond region (within-domain contacts) to the triangle region (across-boundary contacts) around the candidate. Predicted boundaries with *P <.05* were retained, with adjacent retained bins deduplicated by selecting the one with the lower *P*-value. All reported F1 scores are post-Wilcoxon.

### Evaluation framework

Each 25 kb bin was labeled as a boundary (positive) or a non-boundary (negative) according to the reference definition, with all non-boundary bins retained as negatives without subsampling. The positive rate on the test chromosomes ranged from *∼ *0.9% under the filtered definition to *∼ *5.7% under the unfiltered definition, and was *∼ *1.8 to 2.2% for the intersection reference (Supplementary Tables S3 and S4).

Boundary prediction performance was quantified using Precision, Recall, and F1 score. A predicted boundary was counted as a true positive only when its bin matched a reference boundary bin exactly, with no positional tolerance. False positives denote non-boundary bins predicted as boundaries, and false negatives denote reference boundaries missed by the model. Test-set evaluation was conducted on the three held-out chromosomes (chr20, chr21, chr22) across all four cell lines (12 chromosome–cell-line pairs total). Per-cell-line metrics were computed by aggregating true positive, false positive, and false negative counts across the three test chromosomes. Aggregate metrics were computed by pooling counts across all twelve chromosome–cell-line pairs, yielding a single F1 per seed. To assess sensitivity to reference definition, performance was evaluated under all three reference boundary sets (filtered OnTAD, unfiltered OnTAD, and intersection), with the intersection reference adopted as the primary boundary set for cross-architecture comparisons in the main analyses (including LOCO and CTCF orthogonal validation), given that architecture-level comparability was preserved across all three reference definitions.

To quantify seed-level variance, each architecture–hyperparameter configuration was trained with 10 distinct random seeds (123, 42, 0, 7, 100, 1, 2, 3, 8, 13) for within-cell-line experiments and for component ablations. Per-seed F1 scores are reported as mean *± * standard deviation across seeds.

To assess cross-cell-line generalization, LOCO evaluation was performed: for each of the four cell lines, models were trained on the remaining three cell lines (their chr1–15 training chromosomes; chr16, chr17, and chr19 from those three cell lines were used as the validation set) and evaluated on the held-out cell line’s three test chromosomes (chr20–22). LOCO experiments were performed under both default and tuned hyperparameter conditions, with a single random seed (123) per fold; LOCO F1 across the four folds is reported as mean *± * standard deviation across cell lines.

Two robustness analyses were performed, one varying resolution and one varying input coverage. Resolution robustness was assessed by training and evaluating the same architectures, including the parameter-matched controls, at 10 kb resolution, following the identical extraction and KR-balancing protocol. Training chromosomes for which KR balancing failed at 10 kb were excluded identically across all architectures (Supplementary Note S2), so that architecture-level comparisons remain unaffected. Coverage robustness was assessed by downsampling test-set contact matrices via binomial thinning to 50% and 25% of their contact counts, rebalancing by KR normalization, and evaluating with the full-depth models trained under tuned hyperparameters. This probes robustness to reduced input coverage at inference rather than to training at low depth.

In addition, to assess sensitivity to the choice of test chromosomes, three additional chromosome-level splits were evaluated. In each, three chromosomes common to all four cell lines were held out as the test set and three as validation, with the remaining common chromosomes used for training (48 training, 12 validation, and 12 test chromosome–cell-line pairs per split), under default hyperparameters and three seeds.

### CTCF and cohesin enrichment analysis

To assess whether predicted boundaries recover biologically meaningful chromatin features, an orthogonal validation was performed using CTCF binding signal and cohesin (RAD21) occupancy, which are known to colocalize with TAD boundaries [28]. CTCF and RAD21 ChIP-seq signal *P*-value bigWig tracks and IDR-thresholded peaks for the four cell lines (hg38) were obtained from the ENCODE Project [29]. Experiment accessions and file identifiers are listed in Supplementary Table S5.

For each architecture and each cell line, predicted boundaries on the test chromosomes (chr20, chr21, chr22) were obtained from the within-cell tuned-hp model (seed 123) after Wilcoxon post-processing and converted to genomic coordinates at 25 kb resolution, with each boundary center defined as the bin midpoint (bin index *× * 25 kb *+* 12.5 kb). For each predicted boundary, the signal was extracted in a *± 100* kb window around the boundary center at 1 kb resolution using pyBigWig, yielding a 200-bin signal profile per boundary. A boundary-level central signal value was computed as the mean of the central 10 bins (corresponding to *± 5* kb around the boundary center).

Background expectation was estimated by random sampling matched for local sequence and coverage covariates. Fifty independent replicates were generated. In each replicate, random bins on the same test chromosomes were sampled excluding those at predicted boundaries, stratified by per-chromosome contact-coverage decile and GC-content bin so that control bins matched the covariate distribution of predicted boundaries. Gap bins (zero-coverage or high-N regions) were excluded, and the matching caliper was widened where exact matches were unavailable. For each random replicate, the same *± 100* kb signal extraction protocol was applied, and a replicate-level central signal value was computed as the mean of the central 10 bins across all random bins in that replicate.

Fold enrichment was defined per random replicate as the ratio of the mean of boundary-level central signals (averaged across all predicted boundaries) to the corresponding replicate-level central signal. Fold enrichment values are reported as the mean *± * standard deviation across the 50 replicate-level ratios per cell line and architecture. Architecture-level comparisons of fold enrichment were performed using paired *t*-tests across the four cell lines (*n = 4*). Boundary–marker association was additionally quantified by IDR-thresholded peak overlap. For each predicted boundary, overlap of its *± 12.5* kb bin with an IDR peak was recorded, and the fraction of predicted boundaries with such overlap was computed. The same fraction was computed for the matched control bins, and the ratio of the two fractions was reported as the peak-overlap enrichment.

To assess cohesin association at the predicted boundaries, the same enrichment and peak-overlap analyses were performed using RAD21 signal and IDR peaks for all three architectures across the four cell lines.

### Classical TAD caller comparison

AxialTAD was compared against four established non-deep-learning callers on the test chromosomes (chr20–22) at 25 kb, using the same KR-normalized matrices as the main experiments. OnTAD [12] (v1.4) was run with maximum and minimum domain sizes of 200 and 3 bins and a penalty of 0.1. The IS was computed with HiCExplorer [10] (v3.7.6) over depths of 75 to 250 kb. The DI was implemented following Dixon *et al*. [3] over a 2 Mb window, using a threshold-based boundary call rather than the original HMM. TopDom [9] (v0.10.1) was run with a window size of five. Full parameters are given in Supplementary Note S3.

Because OnTAD and the IS were used to construct the intersection reference, the primary comparison assessed CTCF support using IDR-thresholded peaks, rather than independent boundary accuracy. Precision was the fraction of predicted boundary bins containing the midpoint of a CTCF peak, and recall was the fraction of a pooled set of CTCF-supported boundaries recovered within one bin (Supplementary Note S3). Normalized partial AUPRC was calculated over fixed recall interval 0.5607–0.6600 as the area under the linearly interpolated precision–recall curve divided by the interval width. The 95% percentile bootstrap intervals used 1000 resamples of nine cell–chromosome units. K562 was excluded from this benchmark, as CTCF enrichment is absent there for all methods.

As a secondary criterion, intersection-reference F1 was reported for the methods not used to build the reference (DI, TopDom, and the trained models), with a one-bin tolerance. The Wilcoxon rank-sum filter was applied to all methods so that the post-processing pipeline was shared, and both filtered and unfiltered values are reported. Predictions from the trained models were taken from the tuned models at seed 123.

## Results

### Within-cell-line performance and parameter scaling

We evaluated AxialTAD at two configurations (*D=1*, *D=2*) against the deepTAD baseline under two hyperparameter conditions: the *default* setting matching the original deepTAD configuration [21] and the *tuned* setting identified per architecture by validation-driven grid search. The tuned (learning rate, batch size) combinations were $(3 \times 10^{-4}, 256)$ for AxialTAD (*D=1*) and AxialTAD (*D=2*), and $(1 \times 10^{-4}, 256)$ for the deepTAD baseline. The intersection reference (OnTAD *∩ * HiCExplorer) was used as the primary boundary set throughout this section.

AxialTAD in both configurations achieved F1 scores comparable with the deepTAD baseline within the seed-to-seed variation observed across experiments (Table 2). At default hyperparameters, mean F1 across *n = 10* seeds ranged from 0.594 (AxialTAD *D=1*) to 0.608 (deepTAD), with per-seed F1 standard deviations of 0.007–0.020. Under tuned hyperparameters, all three configurations fell within 0.007 F1 of one another (0.596–0.603), well within seed variance. Precision and Recall followed the same comparable pattern across architectures (Precision range 0.495–0.518, Recall range 0.738–0.748 across default and tuned conditions; Table 2). Per-cell-line breakdown confirmed that these F1 values held consistently across all four cell lines—within each cell line, architecture-condition differences remain within 0.02 F1, well below the between-cell-line variation (HeLa-S3 highest, GM12878 lowest; Supplementary Table S6). The comparable pattern across architectures also held under the filtered and unfiltered OnTAD references, although absolute F1 magnitudes differed markedly across reference definitions (filtered *∼ *0.23, intersection *∼ *0.60, unfiltered *∼ * 0.60; Supplementary Table S7). The reduced absolute F1 under the filtered reference reflects the small positive-class count of this highly conservative boundary set (TAD level 1 with score *≥ * 2) rather than degraded architecture performance, as evidenced by the preserved architecture-level comparability under this reference. This comparability was also robust to the boundary-matching rule. Relaxing the exact-match requirement to a tolerance of one or two bins raised F1 by about 0.01 for all architectures, and under one-bin tolerance the difference between AxialTAD and deepTAD was not significant at either depth (Supplementary Table S8).

**Table 2:** Within-cell-line boundary prediction performance under the intersection reference

Architecture	Hp	Precision	Recall	F1
AxialTAD (*D=1*)	default	0.495 *± * 0.032	0.746 *± * 0.040	0.594 *± * 0.020
AxialTAD (*D=1*)	tuned	0.501 *± * 0.033	0.742 *± * 0.040	0.596 *± * 0.011
AxialTAD (*D=2*)	default	0.500 *± * 0.024	0.748 *± * 0.028	0.599 *± * 0.014
AxialTAD (*D=2*)	tuned	0.505 *± * 0.021	0.738 *± * 0.043	0.598 *± * 0.008
deepTAD	default	0.518 *± * 0.020	0.739 *± * 0.027	0.608 *± * 0.007
deepTAD	tuned	0.509 *± * 0.027	0.744 *± * 0.046	0.603 *± * 0.008

Figure 2 shows representative qualitative examples of AxialTAD (*D=2*) and deepTAD predictions on 5 Mb genomic windows from each of the four cell lines, drawn from the held-out test chromosomes (chr20, chr21, chr22). Both architectures recover the majority of reference boundaries with similar prediction patterns across all four cell lines, consistent with the comparable performance reported in Table 2. Across the full set of enumerated windows, per-window recall was strongly correlated between the two architectures (*r = 0.89*), with disagreements concentrated in low-coverage, low-boundary windows where both models under-predict (Supplementary Note S5).

**Figure 2.**
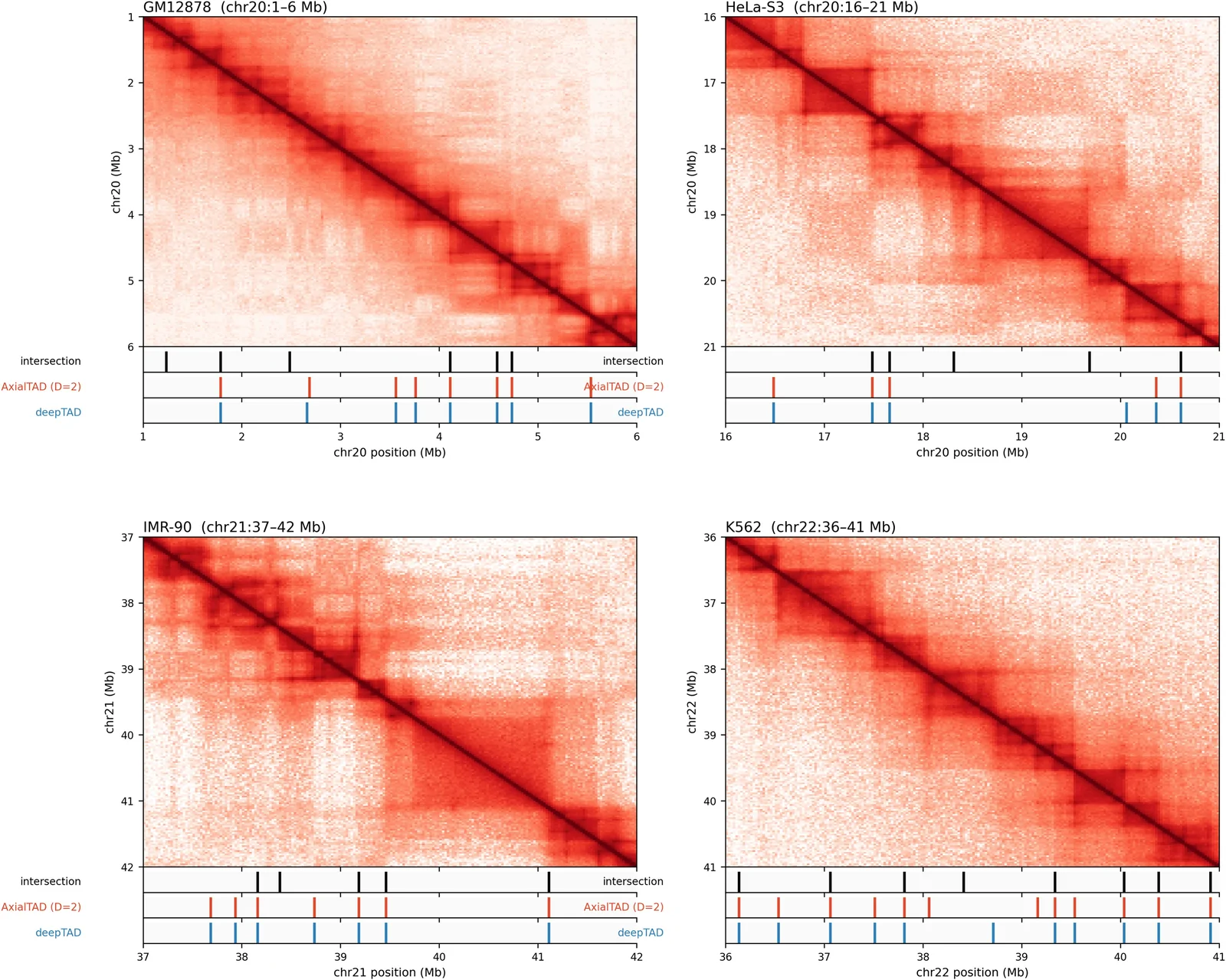
Qualitative comparison of boundary predictions by AxialTAD (*D=2*) and the deepTAD baseline on the held-out test set (intersection reference, tuned hyperparameters, seed 123). One representative 5 Mb window is shown per cell line, selected from the test chromosomes (chr20–22) by a criterion-based procedure detailed in Supplementary Note S4. For each panel, the upper area displays the KR-balanced Hi-C contact matrix at 25 kb resolution; below the matrix, three tracks indicate boundary positions: intersection reference (black), AxialTAD (*D=2*) predictions (red), and deepTAD predictions (blue).

AxialTAD at *D=1* and *D=2* contained 113 356 and 137 420 trainable parameters, respectively—a reduction of 80.1% and 75.9% relative to the deepTAD baseline at *15 × 15* (570 907 parameters; Table 1). AxialTAD thus achieves boundary prediction performance comparable with deepTAD at a substantially smaller model size.

### Architectural ablation and parameter-matched controls

To examine which architectural components of AxialTAD drive performance, we constructed the architectural variants of the primary AxialTAD (*D=2*) configuration with two parameter-matched controls and evaluated them under the same setting (default hyperparameters, intersection reference, *n = 10* random seeds; Table 3). Replacing the entire ACM with a two-stage *1 × 1* convolutional head and center-pixel extraction (ACM-removed) reduced F1 by 0.035 (paired *t*-test against AxialTAD (*D=2*), Holm-adjusted *P =.003*)—the only variant whose change remained significant after multiple-comparison correction. Ablating individual sub-components of the ACM—dropping the vertical-shift branch, dropping the horizontal-shift branch, or removing the sigmoid gating—each produced F1 changes of 0.004 to 0.006, none significant against AxialTAD (*D=2*) (adjusted *P ≥ 0.98*). To probe why dropping either axis branch leaves F1 within seed variance, we quantified the representational overlap between the vertical (Fv) and horizontal (Fh) branch outputs using canonical correlation analysis (Supplementary Note S6, Supplementary Figure S4). Pooled across the four cell lines, the leading held-out canonical correlation between the branches was 0.88, far above a permutation-null 95th percentile of 0.16, indicating that the two branches encode a shared low-dimensional subspace rather than independent information—consistent with either branch alone recovering most of the signal. We also evaluated Transformer-based architectural alternatives at the *15 × 15* patch size to test whether architectures from a different design family yield similar F1: replacing the single combined token with 4, 25, or 100 spatial tokens (multi-token Transformer) yielded F1 differences of 0.004 to 0.006 (adjusted *P = 1.0*), and replacing the multi-head self-attention with a per-token Dense layer (MHA-ablation) yielded a nonsignificant F1 increase of 0.006 (adjusted *P = 1.0*). The mean per-variant seed standard deviation across the eight variants (excluding ACM-removed) was 0.015.

**Table 3:** Component ablation, parameter-matched controls, and Transformer-based architectural variants

Variant	Params	F1	*Δ * versus full	*p* /$p_{\mathrm{adj}}$
**AxialTAD (*D=2*), full**	137 420	0.598 *± * 0.012	—	—
*Parameter-matched controls*				
plain CNN ($3{\times }3$)	137 658	0.597 *± * 0.016	*-0.002*	0.539/1.000
pointwise ($1{\times }1$)	137 408	0.578 *± * 0.022	*-0.020*	0.036/0.320
*ACM component ablations*				
ACM-removed	87 050	0.564 *± * 0.024	*-0.035*	**<0.001**/**0.003**
$F_{v}$ -only	111 211	0.594 *± * 0.010	*-0.004*	0.455/1.000
$F_{h}$ -only	111 211	0.593 *± * 0.014	*-0.006*	0.122/0.979
No-gating	137 354	0.594 *± * 0.014	*-0.004*	0.303/1.000
*Transformer-based variants*				
Multi-token (4)	554 523	0.594 *± * 0.013	*-0.004*	0.151/1.000
Multi-token (25)	898 587	0.593 *± * 0.017	*-0.005*	0.412/1.000
Multi-token (100)	2127 387	0.592 *± * 0.015	*-0.006*	0.247/1.000
MHA-ablation	323 611	0.605 *± * 0.020	*+0.006*	0.205/1.000

To separate the axial form from overall model capacity, we evaluated two parameter-matched controls. The *plain-CNN* control, which replaces the ACM with a standard *3 × 3* convolution, did not differ significantly from AxialTAD ($\Delta F_{1} = -0.002$, 95% CI *[-0.008, +0.004]*, *P =.54*): a parameter-matched local spatial operation achieves the similar in-distribution F1 whether or not it takes the axial form. The pointwise control, which removes spatial mixing while retaining the parameters, fell between AxialTAD and ACM-removed ($\Delta F_{1} = -0.020$, adjusted *P =.32*). Together these indicate that a local spatial operation at the ACM position contributes measurably, but that this operation need not be axial.

By contrast, this pattern was resolution-dependent. At 10 kb resolution, where boundary classification is learned from sparser local context, AxialTAD (*D=2*) exceeded the parameter-matched plain CNN by 0.012 F1 (*P =.022*) and the pointwise control by 0.035 F1 ($P < 10^{-4}$), a wider margin than at 25 kb, where the plain CNN showed similar F1 (Supplementary Table S9). Relative to the four-fold larger deepTAD, the two remained comparable under tuned hyperparameters ($\Delta F_{1} = -0.002$, *P =.79*), while deepTAD’s advantage under default settings ($\Delta F_{1} = -0.027$) tracked its larger parameter count. An advantage over the parameter-matched CNN was also observed in the mouse datasets (Section Evaluation on mouse datasets). Overall, across the variants tested, architectures spanning 87 050 to 2127 387 parameters (Table 1) all fall within *∼ *0.05 F1 of one another at the intersection reference (Table 3), supporting the positioning of AxialTAD as a compact design point.

We also tested whether this similarity depends on the 90$^\circ $ rotation augmentation inherited from the deepTAD protocol. A controlled ablation that separates the rotation effect from the training-data-size confound, using two no-rotation control conditions (Supplementary Note S1, Supplementary Figure S1), showed a small rotation-specific contribution (about 0.016 F1 for AxialTAD), which does not alter the architecture-comparability conclusion.

To assess robustness to input coverage, test matrices were downsampled by binomial thinning to 50% and 25% of their contact counts and rebalanced by KR normalization, and the full-depth models were evaluated on these degraded inputs. F1 declined only modestly (by about 0.03 at 25% depth), and the comparability between AxialTAD and deepTAD was preserved at all depth levels, with the small full-depth difference narrowing at reduced depth (Supplementary Table S10). This assesses robustness to reduced input coverage rather than training at low depth.

### Generalization across cell lines

To assess whether the architectural comparability observed under the within-cell-line evaluation extends to cell lines not seen during training, we performed LOCO evaluation: for each of the four cell lines, AxialTAD (*D=1*, *D=2*) and the deepTAD baseline were trained on the remaining three cell lines and evaluated on the held-out cell, under both default and tuned hyperparameters and the intersection reference.

AxialTAD at both configurations achieved LOCO F1 scores comparable with the deepTAD baseline across all four held-out cell-line folds (Table 4). Under default hyperparameters, mean F1 across the four folds was 0.572 for AxialTAD (*D=1*), 0.570 for AxialTAD (*D=2*), and 0.585 for the deepTAD baseline; pairwise differences (*Δ = -0.015* to *+0.003*) remained within the cell-to-cell standard deviation observed across the four folds (std 0.012 to 0.035). The same pattern held under tuned hyperparameters, with mean F1 of 0.581, 0.571, and 0.588 for the three architectures, respectively. All three architectures showed a modest LOCO drop of 0.017 to 0.033 F1 relative to within-cell-line evaluation, with the magnitude of the drop similar across architectures.

**Table 4:** LOCO F1 scores under the intersection reference

Architecture	Hp	GM12878	HeLa-S3	IMR-90	K562
AxialTAD (*D=1*)	default	0.552	0.622	0.546	0.570
AxialTAD (*D=1*)	tuned	0.554	0.617	0.551	0.601
AxialTAD (*D=2*)	default	0.553	0.579	0.577	0.571
AxialTAD (*D=2*)	tuned	0.535	0.609	0.567	0.573
deepTAD	default	0.566	0.609	0.576	0.587
deepTAD	tuned	0.551	0.600	0.589	0.610

Furthermore, across three additional chromosome-level splits (Supplementary Table S11), deepTAD retained the same small advantage over AxialTAD as in the primary split (at most 0.022 F1, matching in sign and magnitude), indicating that the observed comparability is not specific to the chr20–22 test set.

### CTCF and cohesin enrichment at predicted boundaries

To assess the biological plausibility of predicted boundaries beyond reference-set–based metrics, we computed the enrichment of CTCF ChIP-seq signal around predicted boundary positions. For each architecture and each cell line, CTCF signal intensity (ENCODE *P*-value bigWig tracks; Supplementary Table S5) was aggregated in 1 kb bins over a *± 100* kb window centered on each predicted boundary (post-Wilcoxon, tuned hyperparameters, intersection reference) on the held-out test chromosomes (chr20–22). To control for confounders beyond boundary count, each predicted boundary was compared against random genomic positions matched for local contact coverage and GC content (coverage-decile *× * GC-bin stratified, 50 replicates, gap regions excluded), and boundary–CTCF association was additionally quantified as overlap with IDR-thresholded CTCF peaks relative to matched controls.

AxialTAD (*D=1*, *D=2*) and the deepTAD baseline showed consistent CTCF enrichment at predicted boundaries in three of the four cell lines (Table 5; Supplementary Figure S2; Supplementary Table S12). Fold enrichment ranged from 2.14 to 2.52 across architectures in GM12878, HeLa-S3, and IMR-90, with no apparent architecture-level differences, and predicted boundaries overlapped IDR-thresholded CTCF peaks about twice as often as matched controls in these three cell lines.

**Table 5:** CTCF fold enrichment (signal intensity relative to coverage- and GC-matched control) at predicted boundary positions

Cell line	AxialTAD (*D=1*)	AxialTAD (*D=2*)	deepTAD
GM12878	2.14 *± * 0.32	2.29 *± * 0.26	2.19 *± * 0.32
HeLa-S3	2.21 *± * 0.31	2.19 *± * 0.22	2.26 *± * 0.35
IMR-90	2.52 *± * 0.35	2.42 *± * 0.38	2.46 *± * 0.43
K562	0.94 *± * 0.04	0.94 *± * 0.03	0.93 *± * 0.04

In K562, all three architectures showed near-baseline CTCF signal enrichment (fold *≈ * 0.94) and, in fact, reduced overlap with CTCF peaks relative to matched controls, in contrast to the other three cell lines. Importantly, K562’s within-cell F1 against the caller-derived reference remained *∼ *0.59 across architectures (Supplementary Table S6), despite limited CTCF support. To assess an additional chromatin-associated signal at these predicted positions, we examined cohesin (RAD21) occupancy at the same predicted boundaries using the identical protocol (Supplementary Table S13). In the three CTCF-enriched cell lines, RAD21 recapitulated the CTCF pattern (peak overlap approximately two- to three-fold over control), serving as a positive control. In K562, by contrast, RAD21 remained enriched at predicted boundaries (peak overlap 1.5 to 1.6-fold over control) even as CTCF was depleted. These predicted positions retain cohesin association despite limited CTCF support. This pattern is compatible with CTCF-independent boundary organization, but does not establish that mechanism or independently confirm boundary accuracy. Such CTCF-independent boundary components have been reported in prior work [30, 31], and K562’s status as a cancer-derived, aneuploid line with extensive genome reorganization [32] may contribute to the reduced CTCF association, even though distinguishing the precise mechanisms is beyond the scope of this study.

Across both markers, the enrichment analyses support the predicted boundaries at levels comparable with the deepTAD baseline, with cohesin alignment holding more broadly than CTCF. The comparability observed under the within-cell-line and cross-cell-line settings is thus preserved under this orthogonal biological evaluation.

### Comparison with classical TAD callers

To we compared it against the IS via HiCExplorer [10], the DI [3], and TopDom [9], alongside deepTAD, on the three CTCF-positive cell lines and held-out chromosomes (chr20–22) at 25 kb. OnTAD [12], which was used together with IS to construct the intersection reference, does not expose a continuous operating parameter and could not be swept into a precision–recall curve. It is therefore not included in this comparison.

Because IS contributes to the intersection reference, we compared methods using CTCF support across their precision–recall curves (Supplementary Figure S3; Table 6). This comparison uses external CTCF peaks for scoring and does not measure accuracy against an independent boundary standard.

**Table 6:** Comparison of CTCF support over the restricted recall interval 0.5607–0.6600

Method	Normalized partial AUPRC	95% bootstrap CI
Insulation Score	0.672	[0.619, 0.723]
deepTAD	0.658	[0.619, 0.704]
AxialTAD	0.657	[0.622, 0.695]
TopDom	0.518	[0.455, 0.564]

Normalized partial AUPRC was calculated for GM12878, HeLa-S3, and IMR-90. AxialTAD, deepTAD, and IS had similar point estimates (0.657, 0.658, and 0.672, respectively), while TopDom had a lower estimate (0.518). The bootstrap intervals quantify uncertainty in these estimates and are not interpreted as evidence of statistical equivalence. Threshold sensitivity for AxialTAD and deepTAD is reported in Supplementary Table S14. DI reached a maximum recall of 0.521 and therefore could not be evaluated over the selected interval. For completeness, the same predictions were also scored against the intersection reference with a one-bin tolerance (Supplementary Table S15). Under the shared Wilcoxon post-processing, F1 was 0.310 for the DI and 0.434 for TopDom, against 0.613 for AxialTAD and 0.626 for deepTAD. This criterion favors the trained models by construction, since they were trained on this reference while the classical callers were not, and it is reported to place the absolute F1 values of the main analysis in context rather than as a performance ranking. Low CTCF enrichment in K562 was observed across both classical and learned callers, suggesting that this pattern was not specific to a particular method.

### Evaluation on mouse datasets

Because the axial form conferred no advantage over a parameter-matched CNN within human cell lines at 25 kb (Section Architectural ablation and parameter-matched controls), we compared AxialTAD, a parameter-matched plain CNN, and deepTAD using training and evaluation within each mouse dataset (mESC Micro-C and CH12 Hi-C). Training-set sizes, validation splits, and training settings are detailed in Supplementary Note S7.

On both datasets, AxialTAD achieved higher F1 than the parameter-matched plain CNN (mESC $\Delta F_{1}=+0.111$, CH12 *+0.051*; higher in 19/19 folds for both datasets, $P<10^{-7}$; Table 7). AxialTAD also achieved higher F1 than deepTAD (*+0.045* in mESC and *+0.032* in CH12; $P<10^{-4}$). These results support a benefit of the axial design under the evaluated training settings that is not explained by total parameter count alone.

**Table 7:** Boundary prediction performance under chromosome-level cross-validation within two mouse datasets

	mESC (Micro-C)		CH12 (Hi-C)	
Model	F1	versus plain	F1	versus plain
plain CNN	*0.373 ± 0.045*	—	*0.495 ± 0.039*	—
deepTAD	*0.438 ± 0.050*	*+0.065*	*0.514 ± 0.042*	*+0.019*
AxialTAD	*0.483 ± 0.044*	*+0.111*	*0.546 ± 0.040*	*+0.051*

The advantage observed after training within the mouse datasets did not extend to zero-shot human-to-mouse transfer. Human-trained AxialTAD and deepTAD achieved F1 scores of 0.462 and 0.482 on CH12, respectively, whereas both achieved low F1 on mESC Micro-C (0.013 and 0.026; Supplementary Table S16).

### Computational cost

Parameter count alone does not determine practical efficiency. Training time, inference time, and memory were measured for AxialTAD and deepTAD on identical hardware (single NVIDIA RTX 3090; Supplementary Table S17). Despite its lower parameter count and FLOPs, AxialTAD (*D=2*) required 1.54 times the training time and 1.13 times the inference time of deepTAD, with similar peak GPU memory. AxialTAD (*D=1*) also ran more slowly but used less peak memory (Supplementary Table S17). F1 measured before and after Wilcoxon post-processing showed a small, uniform improvement from filtering (+0.007 to +0.010 across architectures), and the comparability between AxialTAD and deepTAD held both before and after.

## Discussion

We presented AxialTAD, a compact deep learning model for TAD boundary prediction in which the ACM encodes axis-aligned contrast as an explicit inductive bias rather than relying on general-purpose architectural capacity. AxialTAD achieved boundary prediction performance comparable with deepTAD in the human within-cell-line and LOCO evaluations, and higher F1 when trained and evaluated within each mouse dataset.

Taken together, these results indicate that for boundary-centric Hi-C classification at the *15 × 15* patch scale, prediction performance within the range of architectures considered here is not strongly determined by total parameter count. Architectures spanning a 25-fold parameter range fell within *∼ *0.05 F1 of one another at the intersection reference, and the same agreement held in the CTCF analysis, where all three primary architectures showed the same pattern in K562 as in the three CTCF-enriched cell lines. The component ablation results further place the ACM’s contribution at the block level rather than at the level of individual sub-components, since removing the entire ACM produced the only drop that survived multiple-comparison correction while ablating individual axis branches or the gating mechanism remained within seed variance. This pattern is consistent with a partially redundant, complementary design in which multiple internal components encode overlapping boundary-relevant information, and it suggests that designing task-aligned inductive biases is one practical direction for boundary-centric Hi-C classification, complementary to scaling architectural capacity. The augmentation ablation showed a modest contribution of rotation to AxialTAD performance (about 0.016 F1), while deepTAD was largely unaffected. Nevertheless, the F1 difference between the architectures remained within 0.020 without rotation augmentation, indicating that their similar performance was not solely attributable to this augmentation protocol.

The contribution of the axial design was condition-dependent. AxialTAD achieved similar F1 to the parameter-matched CNN in human data at 25 kb, and higher F1 than this control at 10 kb and in both mouse datasets. Although the mouse training sets were smaller, these settings also differ in cell type, assay, resolution, and other data characteristics. The present comparisons do not isolate the factors responsible for the observed advantage and therefore do not establish improved data efficiency.

A methodological observation beyond the architectural conclusions warrants attention: the choice of reference boundary set contributed the single largest effect on F1 in our experiments. Absolute F1 spanned *∼ *0.37 across the filtered, intersection, and unfiltered OnTAD references, an order of magnitude larger than any architecture-level or hyperparameter-level difference (*≤ 0.02* F1). This indicates that reference boundary choice should be treated as a primary methodological consideration in benchmarking TAD boundary prediction methods, since cross-method comparisons using different reference sets can yield apparent performance differences that primarily reflect reference choice rather than architectural merit.

AxialTAD reduces the trainable-parameter count by 76%–80% relative to the deepTAD baseline while achieving similar F1 in the human within-cell-line and LOCO evaluations. For human Hi-C at 25 kb, however, a parameter-matched conventional CNN also achieved similar F1, so these results do not establish a predictive advantage of the axial design in that setting. The reduction in parameter count did not yield a consistent advantage in runtime or peak GPU memory on the tested hardware (Supplementary Table S17). The two mouse datasets provide the main reason to consider AxialTAD, as it outperformed both the parameter-matched CNN and deepTAD. Identifying the data characteristics that favor the axial design would help extend this guidance to other species and assays.

Several limitations should be noted. First, all reference boundaries used for training and evaluation are derived from existing TAD callers (OnTAD and HiCExplorer) and are therefore subject to caller-specific biases. Our multi-reference evaluation (single-caller filtered/unfiltered OnTAD and OnTAD *∩ * HiCExplorer intersection) partially mitigates such biases. The CTCF-support comparison provides complementary evidence of association with a boundary-related factor, but does not constitute an independent assessment of boundary accuracy. We did not directly test whether the model recovers boundaries missed by one caller but supported by independent chromatin evidence, which would require a caller-independent boundary definition that carries its own biases, and we leave this to future work. Second, hyperparameter optimization identified within-cell-line-optimal configurations; cross-cell-line-optimal hyperparameters were not systematically searched, and the within-cell-optimal settings used in the LOCO evaluation may therefore be sub-optimal for cross-cell-line generalization. However, the observed effect of this potential mismatch was small (*≤ 0.013* F1 across architectures). Third, the chr20–22 test set comprises *∼ *162 Mb of genomic sequence, which limits test-set statistical power, particularly for per-cell-line comparisons and Wilcoxon-based post-processing. Three additional chromosome-level splits reproduced the same architecture-level pattern, but each is subject to the same constraint. Fourth, absolute F1 values across architectures remained around 0.60 under the intersection reference, reflecting the inherent difficulty of TAD boundary prediction and the noise in caller-derived labels.

The present analysis characterizes the ACM’s contribution through component ablation. A finer mechanistic account of how individual sub-components encode boundary-relevant patterns would require attribution methods beyond aggregate performance, which we leave to future work.

Despite these limitations, the central finding held under multiple reference boundary definitions, hyperparameter conditions, and augmentation protocols. Explicit axis-aligned contrast modeling matched a CNN+Transformer baseline at *∼ *20% of the parameter count within distribution, while conferring a measurable advantage when trained and evaluated within the two mouse datasets, supporting AxialTAD as a compact design point for boundary-centric Hi-C classification. Future work could extend the comparisons with conventional TAD callers to a broader range of resolutions, species, and assays to clarify which data characteristics favor the axial design. Feature visualization and attribution analyses, together with additional epigenomic data such as active histone modifications, could further clarify how the ACM captures boundary-associated patterns and help interpret predicted boundaries with limited CTCF support.

**Key Points**

- We propose AxialTAD, a deep learning model for TAD boundary prediction centered on an Axial Contrast Module (ACM) that explicitly encodes directional contrast along the row and column axes, motivated by the geometry of TAD boundaries.
- AxialTAD achieves boundary prediction performance comparable with a recent CNN+Transformer baseline, while using 76%–80% fewer trainable parameters (113K–137K versus 571K).
- CTCF and cohesin ChIP-seq analyses provided complementary biological evidence for boundaries predicted by the within-cell-line models.
- The benefit of the explicit axial form was condition-dependent, with AxialTAD performing similarly to a parameter-matched CNN in human Hi-C at 25 kb and achieving higher F1 within each of two mouse datasets.
- Component ablation places the ACM’s contribution at the block level: removing the whole module produces the largest F1 change, while ablating individual sub-components stays within seed-level variance.

## Supplementary Material

AxialTAD_Suppl_R2_bbag603

## Data Availability

The source codes underlying this article are available at https://github.com/Biomedical-Data-Science-Laboratory/AxialTAD.
