# Supplementary material for: AxialTAD: an axial contrast architecture for TAD boundary prediction from Hi-C
Source: Brief Bioinform. 2026 Nov 2;27(6):bbag603. doi: 10.1093/bib/bbag603 (PMC13725924; doi:10.1093/bib/bbag603)
Supplement: Supplementary file 1 [file axialtad_suppl_r2_bbag603.pdf]

AxialTAD: An Axial Contrast Architecture for TAD Boundary Prediction from Hi-C

Supplementary Material

Supplementary Notes

Supplementary Note S1. Augmentation ablation: B1/B2 control protocol

The augmentation ablation decomposes the effect of rotation augmentation into two components, an orientation prior and a training-data-size effect, by comparing the with-augmentation baseline against two no-rotation controls, B1 and B2.

Motivation Rotation augmentation has two effects that a naive no-rotation ablation would confound. First, it acts as an orientation prior: each positive training submatrix is rotated by 90° and the rotated copy is retained as an additional training example, encouraging representations robust to the corresponding change in patch orientation. Second, it doubles the positive count, which expands the negative pool (the negative-to-positive cap is defined on the post-augmentation positive count); disabling rotation therefore also reduces per-epoch training-data size.

Rotation geometry Because each patch is a symmetric block of the contact matrix, a 90° clockwise rotation is equivalent to a left–right mirror of the patch, and reorients the contact block from the main diagonal onto the anti-diagonal. The rotated patches therefore do not correspond to configurations that occur in observed contact matrices. The transformation is label-preserving, since the boundary status of the central bin is unchanged. A single 90° rotation is used, rather than the full 90°/180°/270° set, to follow the augmentation protocol of deepTAD [1] for direct comparability.

Control conditions In B1, rotation is disabled and the negative-to-positive ratio is held at the baseline 4:1. Because rotation is the main mechanism expanding the negative pool, this reduces per-epoch training-data size to about 50% of the with-augmentation condition, so B1 confounds the orientation-prior effect with a data-size reduction and reflects their joint effect. In B2, rotation is disabled and the ratio is doubled to 8:1 by sampling additional negatives uniformly from the candidate pool. This restores per-epoch training-data size to about 90% (the residual gap reflects the absent rotated positives, which a negative-ratio adjustment cannot compensate), so B2 isolates the orientation-prior effect at approximately matched data size, with the residual mismatch acting to slightly understate any rotation-specific benefit.

Protocol For each of the three primary architectures (AxialTAD $D = 1$, AxialTAD $D = 2$, deepTAD) and each condition (with-augmentation, B1, B2), models were trained at both the architecture's tuned hyperparameters (Table S2) and the default hyperparameters, each with four

seeds from the main seed bank. The chromosome-level split was held fixed. Reported F1 is post-Wilcoxon, averaged over seeds. Per-condition ΔF_1 is computed within each (architecture, hyper-parameter) pair as $\Delta F_1 = F_1(\text{condition}) - F_1(\text{baseline})$ and pooled across the two hyperparameter conditions, giving $n = 8$ measurements per architecture per condition.

Results Under B1, all three architectures lost comparable F1 ($\Delta F_1 \approx -0.075$ to -0.078; s.d. 0.015–0.020), with no resolvable cross-architecture difference (paired t-test, $p \geq 0.68$). The dominant effect at B1 is therefore the data-size reduction rather than architecture-specific sensitivity to rotation. Under B2, AxialTAD ($D = 1$, $D = 2$) lost modest F1 (-0.016 ± 0.016 and -0.017 ± 0.021) while deepTAD was essentially unchanged ($+0.001 \pm 0.011$), a small but resolvable difference (AxialTAD $D = 1$ vs deepTAD $p = 0.015$; $D = 2$ vs deepTAD $p = 0.014$). At matched data size AxialTAD therefore loses about 0.016 F1 without rotation while deepTAD does not.

Interpretation The axial operators are explicitly aligned with the row and column axes, so a change in patch orientation alters the signed-difference profile, whereas deepTAD's spatial-attention head re-weights features from a learned 2D map and is largely orientation-robust by construction. The rotation-specific effect (≈ -0.016 F1 for AxialTAD at B2) is modest relative to other sources of variation (within-cell architecture-condition differences ≈ 0.005; cross-cell-line ≈ 0.02–0.05; cross-reference ≈ 0.37), and does not change the within-cell comparability conclusion. The decomposition also shows that training-data-size control is essential in this task, since a naive no-rotation comparison that does not match data size overstates the rotation-specific contribution by about 0.06 F1.

Supplementary Note S2. Resolution robustness: 10 kb preprocessing

At 10 kb resolution, KR balancing additionally failed for IMR-90 chr7 and K562 chr1, chr2, and chr4 (about 7% of training chromosomes). These were excluded uniformly across all four architectures (AxialTAD $D = 2$, plain CNN, pointwise, deepTAD), so architecture-level differences are not confounded by differing training data. All test chromosomes (chr20–22) passed KR balancing for all four cell lines.

Supplementary Note S3. Classical TAD caller comparison

This section provides the full parameter settings and evaluation details for the classical caller comparison summarized in the main text.

Additional caller parameters Beyond the settings given in the main text, OnTAD [2] used log-likelihood parameters `ldiff` 1.96 and `lsize` 5, with boundaries taken unfiltered at level one or above. The Insulation Score (HiCExplorer [3]) used a step of 25 kb with FDR-corrected thresholding at 0.01. The Directionality Index [4] used an adaptive per-chromosome threshold at the 70th percentile of the absolute DI distribution, with sign changes required within three bins. Because this implementation uses threshold-based calling rather than the original hidden Markov model, it yields sparser boundary sets than the other methods. TopDom [5] was run under R 4.3.3.

Evaluation details For precision, a predicted boundary was counted as CTCF-supported if its own 25 kb bin contained the midpoint of an IDR-thresholded CTCF peak. No additional positional tolerance was applied. The recall pool was constructed from CTCF-supported boundaries at the native operating points of seven methods: AxialTAD ($D = 1$ and $D = 2$), deepTAD, OnTAD, IS, DI, and TopDom. Pool boundaries were merged within one bin, and recall matching also used a one-bin tolerance. All boundaries were Wilcoxon-filtered before scoring.

Operating points All boundary sets were Wilcoxon-filtered before scoring. AxialTAD ($D = 2$) and deepTAD used probability thresholds from 0.05 to 0.35 in increments of 0.025, followed by 0.4, 0.5, 0.6, 0.7, and 0.8 (18 thresholds per model). For IS, the FDR-corrected `--thresholdComparisons` values were 0.00075, 0.001, 0.0015, 0.002, 0.003, 0.004, 0.005, 0.01, and 0.05. Five stricter values (10^{-5}, 5×10^{-5}, 10^{-4}, 2.5×10^{-4}, and 5×10^{-4}) were evaluated but yielded no boundaries across the nine evaluated cell–chromosome units (GM12878, HeLa-S3, and IMR-90; chr20–22). An operating point was omitted when the total number of post-Wilcoxon predictions across the evaluated units was zero, making precision undefined. This rule was applied to both the full-data curves and each bootstrap replicate. TopDom window sizes were 5, 8, 10, 12, 15, 20, 25, 30, and 40 bins. DI thresholds were the 30th, 35th, 40th, 45th, 50th, 55th, 60th, 65th, 70th, and 80th percentiles of the per-chromosome |DI| distribution.

Recall interval The recall interval was fixed at 0.5607–0.6600, using the limits selected in the original analysis. Additional operating points were evaluated while retaining this interval, the recall pool, matching rules, and Wilcoxon post-processing. Precision was linearly interpolated onto a 50-point recall grid. Normalized partial AUPRC was calculated by trapezoidal integration over this grid and division by the interval width. Operating points with no predicted boundaries were omitted because precision was undefined. DI did not reach the selected interval.

Confidence intervals Block bootstrap resampling used nine cell–chromosome units (three cell lines by three chromosomes), with the same resampled units used across methods in each of 1,000 replicates. The recall interval and scoring rules were held fixed. With the expanded operating-point sweeps, all four methods reported in Table 6 covered the full interval in every replicate; no extrapolation or resample exclusion was used. The reported intervals are the 2.5th and 97.5th percentiles of the bootstrap estimates. Overlapping intervals are not interpreted as evidence of statistical equivalence. For IS, some resampled curves covered the lower end of the interval through linear interpolation from a more distant low-recall operating point, so the estimates remain dependent on the interpolation between discrete operating points.

Supplementary Note S4. Region selection for Figure 2

The qualitative comparison in main-text Figure 2 displays one representative 5 Mb window per cell line, drawn from the held-out test chromosomes (chr20–22). To avoid the bias of manual selection, regions were chosen by an algorithmic, criterion-based procedure designed to display windows that reflect typical model behavior in each cell line rather than best- or worst-case examples.

Enumeration For each cell line and each test chromosome, all 5 Mb windows were enumerated in 1 Mb steps. Each window was characterized by its reference boundary count under the intersection definition, the per-window recall of AxialTAD ($D = 2$) and deepTAD at tuned hyperparameters (seed 123, post-Wilcoxon), and the zero-row fraction of the KR-balanced matrix restricted to the window, used as a proxy for local data sparsity.

Filtering Windows were retained when they satisfied all of the following: a reference boundary count between 5 and 12 (sufficient boundaries for inspection without saturation); an absolute recall difference between the two architectures of at most 0.15 (so the window is informative about architecture-level comparison rather than an isolated discordance); a zero-row fraction of at most 5% (excluding regions where KR-balancing failure obscures TAD structure); and a per-window recall within ±15% of the cell line's mean per-window recall (so the window reflects typical behavior). The candidate pools after filtering contained 10, 29, 22, and 35 windows for GM12878, HeLa-S3, IMR-90, and K562, respectively.

Joint selection From these pools, the four displayed regions were selected jointly by an exhaustive search over a composite score favoring recall diversity across the four panels, with coverage of multiple test chromosomes and low zero-row fraction as tie-breakers. The scoring function and the full enumerated pool are provided in the accompanying repository.

Regions selected for the qualitative comparison

Cell line	Region	Boundaries	AxialTAD R	deepTAD R
GM12878	chr20: 1–6 Mb	6	0.667	0.667
HeLa-S3	chr20: 16–21 Mb	5	0.600	0.600
IMR-90	chr21: 37–42 Mb	5	0.800	0.800
K562	chr22: 36–41 Mb	8	0.875	0.875

The selected regions span three test chromosomes. In every selected region AxialTAD ($D = 2$) and deepTAD recovered the same reference boundaries, consistent with the within-cell architectural comparability reported in main-text Table 2. Because per-window recall is discrete at these small boundary counts, equal recall in the four panels reflects this comparability rather than selection toward matched architectures; the retained candidate pools span a substantially wider recall range than the four displayed values.

Supplementary Note S5. Architecture agreement and failure modes across the full window set

Beyond the four displayed regions, we examined all 5 Mb windows enumerated across the four cell lines and test chromosomes (592 windows; 502 containing at least one reference boundary). Per-window recall was strongly correlated between AxialTAD ($D = 2$) and deepTAD (Pearson $r = 0.89$), and the two architectures recovered identical boundaries in a majority of windows (median $|\Delta\text{recall}| = 0$). Windows with large per-window disagreement ($|\Delta\text{recall}| > 0.15$; 20% of scored windows) were concentrated almost entirely in windows containing very few reference boundaries (one to three), where a single missed boundary shifts recall between 0 and 1; the five largest-$|\Delta\text{recall}|$ windows all had one to three boundaries. This disagreement therefore reflects the discreteness of per-window recall at low boundary counts rather than a systematic architecture-level difference. Recall for both architectures declined together in the harder windows: in low-coverage windows (zero-row fraction > 0.10) mean recall fell to 0.33 (AxialTAD) and 0.30 (deepTAD), versus 0.75 for both in well-covered windows, and in windows with at most two reference boundaries recall averaged about 0.48 for both, versus about 0.75 in windows with three or more. Low-coverage and low-boundary windows overlap substantially, and the between-architecture recall difference in these windows was if anything smaller (mean $|\Delta\text{recall}|$ 0.058) than in the full set (0.071). The dominant failure mode is thus shared by both architectures and driven by local data sparsity—few contacts and few boundaries per window—rather than being specific to either architecture, consistent with the region-selection criterion (five to twelve boundaries, zero-row fraction $\leq 5\%$) used for the qualitative comparison in Figure 2.

Supplementary Note S6. Axis-branch redundancy (canonical correlation analysis)

Feature extraction We extracted the vertical-branch (Fv) and horizontal-branch (Fh) outputs at the center coordinate of the 5×5 feature map (32 channels each) for AxialTAD ($D = 2$) at tuned hyperparameters (seed 123, intersection reference) on the held-out test patches (chr20–22). Both the pre-gating branch outputs (Eq. 5) and the post-gating features entering the fusion step were analyzed.

Canonical correlation and null Because canonical correlation analysis (CCA) overestimates correlation in this dimension-to-sample regime (the in-sample leading correlation was 0.95–1.00 across all conditions), canonical directions were fit on one random half of the patches and correlations evaluated on the held-out half. A permutation null was constructed by independently row-permuting the channels of Fh to break the Fv–Fh sample pairing (200 iterations, identical held-out procedure); the 95th percentile across iterations defines the null band. Pure channel-index permutation leaves CCA invariant and was therefore not used. Channel-wise cross-correlation was additionally computed as the 32×32 matrix of Pearson correlations between individual Fv and Fh channels.

Results Pooled across the four cell lines (499 boundary patches, with an equal-sized non-boundary subsample), the leading held-out canonical correlation between the branches was 0.88 pre-gating and 0.83 post-gating, with the top five components all exceeding the null 95th percentile (0.16 and 0.18 for the leading component, respectively). This overlap was present in both boundary and non-boundary patches (leading held-out correlation 0.88 versus 0.89 pre-gating), indicating a structural redundancy between the branches, expected as both derive from the same encoder output, rather than a boundary-specific coupling. The channel-wise cross-correlation was low (mean $|r| \approx 0.11$): the redundancy is a shared low-dimensional subspace captured by basis-free CCA, not a one-to-one channel correspondence. Per-cell-line estimates were underpowered ($\approx$120 boundary patches per line) and are reported as secondary; post-gating variants and per-cell-line results are provided in the repository.

Interpretation Because the two branches carry largely overlapping information, removing either one leaves the other able to recover most of the boundary signal, whereas removing the entire ACM removes the shared subspace itself.

Supplementary Note S7. Evaluation within mouse datasets

Data splits and training-set sizes Models were trained and evaluated separately within mESC Micro-C and CH12-LX in situ Hi-C using 19-fold leave-one-chromosome-out cross-validation over chr1–19. For test chromosome k, chromosome $(k \bmod 19) + 1$ was used for validation and the remaining 17 chromosomes for training. No autosomes were excluded. Before augmentation, training folds contained 1,574–1,760 positive bins in mESC and 1,479–1,604 in CH12. After 90-degree rotation augmentation of positive samples and negative subsampling at a 4:1 negative-to-positive ratio, the training sets contained 15,740–17,600 and 14,790–16,040 patches per fold, respectively. The pooled four-cell-line human training set contained 71,780 patches after the same processing.

Training settings Each fold was trained with three seeds (123, 42, and 0) for 30 epochs, retaining the checkpoint with the highest validation F1. Batch size was 256. Learning rates were those selected in the human experiments: 3×10^{-4} for AxialTAD and 1×10^{-4} for deepTAD. The parameter-matched plain CNN used the same learning rate as AxialTAD. No mouse-specific hyperparameter tuning was performed.

Evaluation and statistical analysis For Table 7, post-Wilcoxon F1 under exact-bin matching was first averaged across the three seeds within each held-out chromosome. Results are the unweighted mean ± SD of these seed-averaged scores across 19 folds. Architectures were compared using two-sided paired t-tests on the seed-averaged fold scores. Separately, human-trained models were evaluated on both mouse datasets without retraining, fine-tuning, or threshold adjustment to assess zero-shot human-to-mouse transfer (Supplementary Table S16). The models were trained on the pooled four-cell-line human training set. F1 was calculated after pooling TP, FP, and FN across the 19 mouse autosomes for each of ten training seeds, and is reported as the mean ± SD across seeds.

References

[1] Xiaoyan Wang, Junwei Luo, Lili Wu, Huimin Luo, and Fei Guo. deepTAD: an approach for identifying topologically associated domains based on convolutional neural network and transformer model. *Briefings in Bioinformatics*, 26(2):bbaf127, 03 2025.

[2] Lin An, Tao Yang, Jiahao Yang, Johannes Nuebler, Guanjue Xiang, Ross C. Hardison, Qunhua Li, and Yu Zhang. OnTAD: hierarchical domain structure reveals the divergence of activity among TADs and boundaries. *Genome Biology*, 20(1):282, 2019.

[3] Fidel Ramírez, Vivek Bhardwaj, Laura Arrigoni, Kin Chung Lam, Björn A. Grüning, José Villaveces, Bianca Habermann, Asifa Akhtar, and Thomas Manke. High-resolution TADs reveal DNA sequences underlying genome organization in flies. *Nature Communications*, 9(1):189, 2018.

[4] Jesse R. Dixon, Siddarth Selvaraj, Feng Yue, Audrey Kim, Yan Li, Yin Shen, Ming Hu, Jun S. Liu, and Bing Ren. Topological domains in mammalian genomes identified by analysis of chromatin interactions. *Nature*, 485(7398):376–380, 2012.

[5] Hanjun Shin, Yi Shi, Chao Dai, Harianto Tjong, Ke Gong, Frank Alber, and Xianghong Jasmine Zhou. TopDom: an efficient and deterministic method for identifying topological domains in genomes. *Nucleic Acids Research*, 44(7):e70, 2016.

Supplementary Tables

Table S1: Hyperparameter grid evaluated during validation-driven tuning.

lr \ batch	64	128	256	512	1024
3×10^{-5}			✓	✓	✓
1×10^{-4}	✓	✓	✓	✓	✓
3×10^{-4}	✓	✓	✓	✓	✓
1×10^{-3}	✓	✓	✓		

Table S2: Architecture-specific tuned hyperparameters selected by best validation F1.

Architecture	Tuned lr	Tuned batch	Best val F1	Best epoch
AxialTAD (D=1)	3×10^{-4}	256	0.609	23
AxialTAD (D=2)	3×10^{-4}	256	0.597	22
deepTAD	1×10^{-4}	256	0.626	20

Table S3: Positive sample counts (boundary bins) on the test chromosomes for each cell line and reference definition. Percentages are relative to all candidate bins on the three test chromosomes.

Cell line	Reference	chr20	chr21	chr22	Total (%)
GM12878	filtered	29	13	19	61 (0.94)
	intersection	55	20	44	119 (1.84)
	unfiltered	140	68	109	317 (4.89)
HeLa-S3	filtered	38	20	25	83 (1.28)
	intersection	62	35	42	139 (2.15)
	unfiltered	164	89	113	366 (5.65)
IMR-90	filtered	30	16	24	70 (1.08)
	intersection	56	21	43	120 (1.85)
	unfiltered	149	77	108	334 (5.15)
K562	filtered	28	13	15	56 (0.86)
	intersection	52	23	46	121 (1.87)
	unfiltered	156	77	110	343 (5.29)

Table S4: Positive sample counts (boundary bins) in the training and validation sets for each cell line and reference definition.

Cell line	Reference	Train pos. (%)	Val pos. (%)
GM12878	filtered	721 (0.86)	108 (1.16)
	intersection	1,506 (1.79)	169 (1.82)
	unfiltered	4,224 (5.01)	527 (5.67)
HeLa-S3	filtered	1,239 (1.29)	135 (1.45)
	intersection	2,219 (2.31)	217 (2.34)
	unfiltered	5,456 (5.68)	570 (6.14)
IMR-90	filtered	1,099 (1.14)	131 (1.41)
	intersection	1,876 (1.95)	198 (2.13)
	unfiltered	4,867 (5.07)	544 (5.86)
K562	filtered	802 (0.95)	118 (1.27)
	intersection	1,577 (1.87)	220 (2.37)
	unfiltered	4,234 (5.03)	589 (6.34)

Table S5: ENCODE CTCF and RAD21 ChIP-seq accessions used for orthogonal validation.

Target	Cell line	Experiment	Signal bigWig	Peaks
CTCF	GM12878	ENCSR000DZN	ENCFF637RGD	ENCFF138REW
	HeLa-S3	ENCSR000AOA	ENCFF152FJU	ENCFF111RWV
	IMR-90	ENCSR000EFI	ENCFF101PSS	ENCFF670ULH
	K562	ENCSR000BPK	ENCFF719NSK	ENCFF039IZP
RAD21	GM12878	ENCSR000EAC	ENCFF820XGC	ENCFF004RJL
	HeLa-S3	ENCSR000EDE	ENCFF602HAP	ENCFF331IAD
	IMR-90	ENCSR000EFJ	ENCFF597FBQ	ENCFF395SJE
	K562	ENCSR942XQI	ENCFF573KKU	ENCFF710DZU

Table S6: Per-cell-line within-cell F1 (intersection reference, ten seeds).

Architecture	Hp	GM12878	HeLa-S3	IMR-90	K562
AxialTAD (D=1)	default	0.559 ± 0.028	0.623 ± 0.021	0.610 ± 0.025	0.583 ± 0.022
AxialTAD (D=1)	tuned	0.558 ± 0.015	0.626 ± 0.026	0.612 ± 0.009	0.587 ± 0.011
AxialTAD (D=2)	default	0.561 ± 0.022	0.630 ± 0.020	0.611 ± 0.018	0.591 ± 0.014
AxialTAD (D=2)	tuned	0.569 ± 0.018	0.623 ± 0.012	0.610 ± 0.016	0.591 ± 0.009
deepTAD	default	0.576 ± 0.013	0.642 ± 0.013	0.619 ± 0.014	0.596 ± 0.012
deepTAD	tuned	0.573 ± 0.011	0.630 ± 0.016	0.615 ± 0.015	0.593 ± 0.013

Table S7: Within-cell F1 across three reference boundary definitions (ten seeds).

Architecture (hp)	filtered	intersection	unfiltered
AxialTAD (D=1) default	0.223 ± 0.018	0.594 ± 0.020	0.591 ± 0.009
AxialTAD (D=1) tuned	0.239 ± 0.016	0.596 ± 0.011	0.597 ± 0.010
AxialTAD (D=2) default	0.228 ± 0.015	0.599 ± 0.014	0.593 ± 0.014
AxialTAD (D=2) tuned	0.241 ± 0.013	0.598 ± 0.008	0.599 ± 0.006
deepTAD default	0.230 ± 0.019	0.608 ± 0.007	0.598 ± 0.011
deepTAD tuned	0.230 ± 0.018	0.603 ± 0.008	0.596 ± 0.008

Table S8: F1 under matching tolerances of zero, one, and two bins (within-cell, intersection reference, ten seeds).

Architecture	tol. 0	tol. 1	tol. 2
AxialTAD (D=1)	0.596 ± 0.011	0.607 ± 0.010	0.608 ± 0.010
AxialTAD (D=2)	0.598 ± 0.008	0.609 ± 0.008	0.610 ± 0.008
deepTAD	0.603 ± 0.008	0.612 ± 0.006	0.613 ± 0.006

Table S9: Boundary prediction F1 at 10 kb resolution.

Architecture	F1 (default)	F1 (tuned)	Params
plain CNN	0.498 ± 0.034	—	137,658
pointwise	0.475 ± 0.034	—	137,408
AxialTAD (D=2)	0.510 ± 0.029	0.500 ± 0.032	137,420
deepTAD	0.537 ± 0.021	0.502 ± 0.031	570,907

Table S10: F1 under input-coverage downsampling.

Architecture	full	50%	25%
AxialTAD (D=1)	0.596 ± 0.011	0.587 ± 0.013	0.567 ± 0.009
AxialTAD (D=2)	0.598 ± 0.008	0.587 ± 0.009	0.567 ± 0.008
deepTAD	0.603 ± 0.008	0.590 ± 0.010	0.569 ± 0.010

Table S11: Human chromosome-level cross-validation F1 across four held-out chromosome splits (default hyperparameters, intersection reference, three seeds). ΔF1 is the difference from deepTAD

Fold	Test chrs	AxialTAD (D=1)	AxialTAD (D=2)	deepTAD	ΔF1 (D=1)	ΔF1 (D=2)
0	20,21,22	0.595 ± 0.014	0.603 ± 0.007	0.607 ± 0.001	−0.013	−0.004
A	13,14,15	0.600 ± 0.029	0.595 ± 0.016	0.617 ± 0.005	−0.018	−0.022
B	16,17,19	0.582 ± 0.016	0.577 ± 0.005	0.590 ± 0.015	−0.008	−0.013
C	4,5,6	0.592 ± 0.006	0.604 ± 0.013	0.598 ± 0.013	−0.006	+0.006

Table S12: CTCF fold enrichment at predicted boundaries (coverage- and GC-matched control, 50 replicates). n_{pred} is the number of predicted boundaries retained after Wilcoxon post-processing.

Architecture	Cell line	Fold (mean ± sd)	n_{pred}
AxialTAD (D=1)	GM12878	2.139 ± 0.322	180
	HeLa-S3	2.210 ± 0.309	168
	IMR-90	2.522 ± 0.352	174
	K562	0.941 ± 0.035	174
AxialTAD (D=2)	GM12878	2.290 ± 0.259	179
	HeLa-S3	2.192 ± 0.223	171
	IMR-90	2.423 ± 0.382	172
	K562	0.944 ± 0.033	189
deepTAD	GM12878	2.189 ± 0.319	178
	HeLa-S3	2.261 ± 0.353	161
	IMR-90	2.459 ± 0.426	162
	K562	0.932 ± 0.039	169

Table S13: CTCF and RAD21 peak-overlap enrichment at predicted boundaries. Peak-overlap fractions were averaged across chromosomes. Overlap was defined between the predicted boundary bin and the peak interval, without additional positional tolerance.

Target	Cell line	AxialTAD (D=1)	AxialTAD (D=2)	deepTAD
CTCF	GM12878	1.89	1.99	1.94
	HeLa-S3	2.00	2.00	1.99
	IMR-90	2.12	2.16	2.23
	K562	0.53	0.62	0.61
RAD21	GM12878	2.70	3.01	2.83
	HeLa-S3	2.18	2.27	2.19
	IMR-90	2.15	2.12	2.22
	K562	1.50	1.60	1.50

Table S14: Threshold sensitivity of AxialTAD ($D = 2$) and deepTAD on the CTCF-support criterion, pooled over the three CTCF-positive cell lines. Count is the mean number of predicted boundaries per cell line, and $F1_h$ the harmonic mean of the CTCF-alignment precision and recall.

Threshold	Count	Precision	Recall	$F1_h$
AxialTAD (D=2)				
0.8	97.7	0.758	0.268	0.396
0.7	124.3	0.732	0.336	0.461
0.6	154.0	0.699	0.408	0.515
0.5	174.0	0.682	0.458	0.548
0.4	194.7	0.682	0.511	0.584
0.3	224.3	0.660	0.581	0.618
deepTAD				
0.8	86.3	0.757	0.244	0.369
0.7	116.7	0.726	0.322	0.446
0.6	143.3	0.707	0.389	0.502
0.5	167.0	0.689	0.448	0.543
0.4	193.3	0.681	0.514	0.586
0.3	233.0	0.657	0.608	0.631

Table S15: Intersection-reference F1 for methods not used to construct the reference, pooled across four cell lines and the three test chromosomes with a one-bin matching tolerance.

Method	Post-processing	Precision	Recall	F1
Directionality Index	none	0.341	0.275	0.304
	Wilcoxon	0.387	0.259	0.310
TopDom	none	0.225	0.950	0.363
	Wilcoxon	0.294	0.830	0.434
AxialTAD ($D = 2$)	none	0.484	0.794	0.601
	Wilcoxon	0.522	0.744	0.613
deepTAD	none	0.509	0.782	0.616
	Wilcoxon	0.546	0.734	0.626

Table S16: Zero-shot transfer from human-trained models to mouse.

Model	mESC F1	CH12 F1
AxialTAD (D=2)	0.013 ± 0.018	0.462 ± 0.074
deepTAD	0.026 ± 0.036	0.482 ± 0.049

Table S17: Computational cost of AxialTAD and deepTAD (single NVIDIA RTX 3090). Training time covers 30 epochs on 71,780 patches. Inference time covers model prediction on 25,920 test patches, excluding data loading and Wilcoxon post-processing. Both used batch size 256. The times were averaged over three training runs after warm-up and five inference runs. Peak memory is the TensorFlow allocator peak during one training run.

	AxialTAD (D=1)	AxialTAD (D=2)	deepTAD
Parameters	113,356	137,420	570,907
FLOPs (per patch)	5.65M	6.87M	8.74M
GPU training (s)	42.3	46.0	29.8
GPU inference (s)	0.247	0.267	0.235
GPU peak memory (MB)	148	183	182

Supplementary Figures

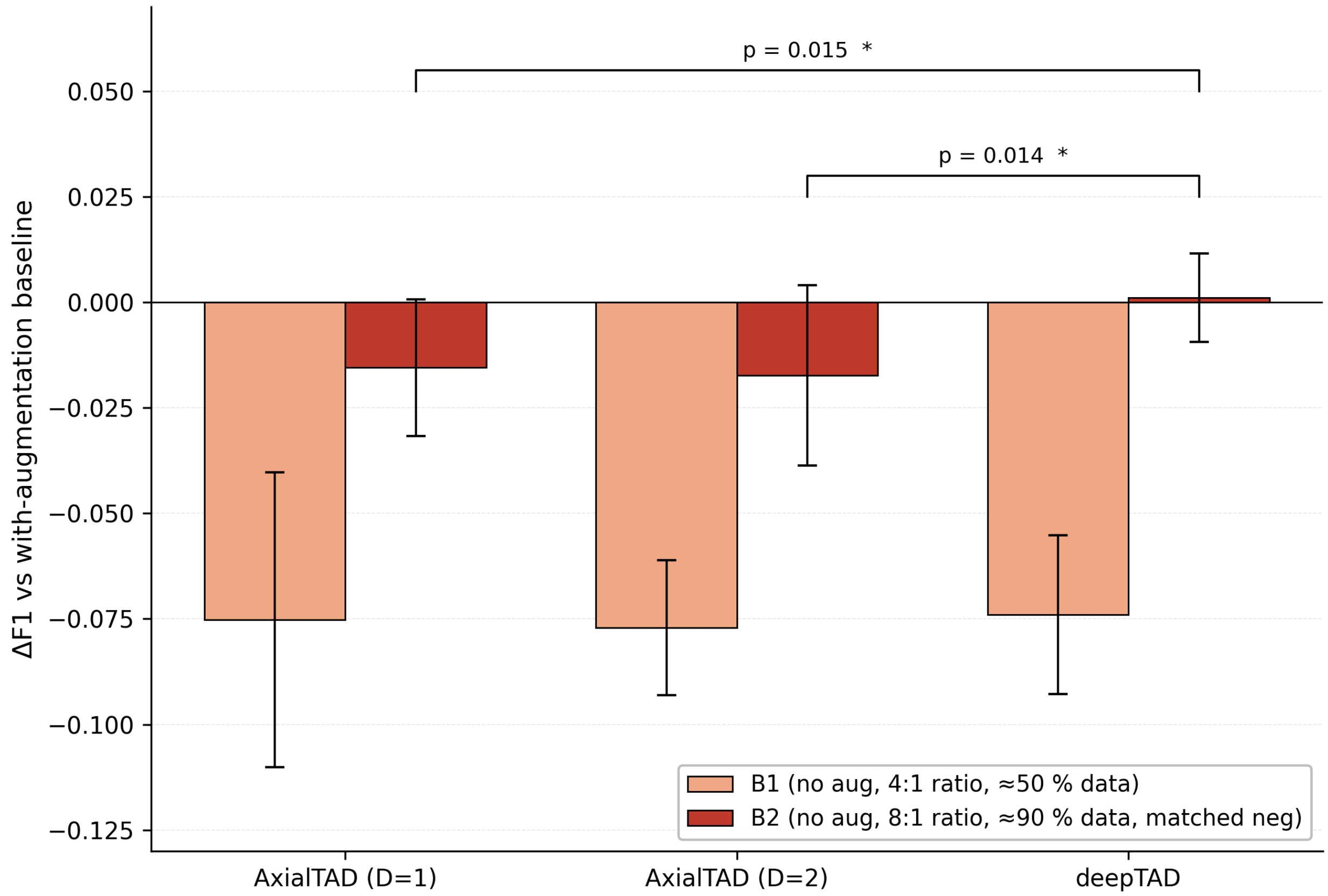

Figure S1: Augmentation ablation (B1/B2) ΔF_1 by architecture.

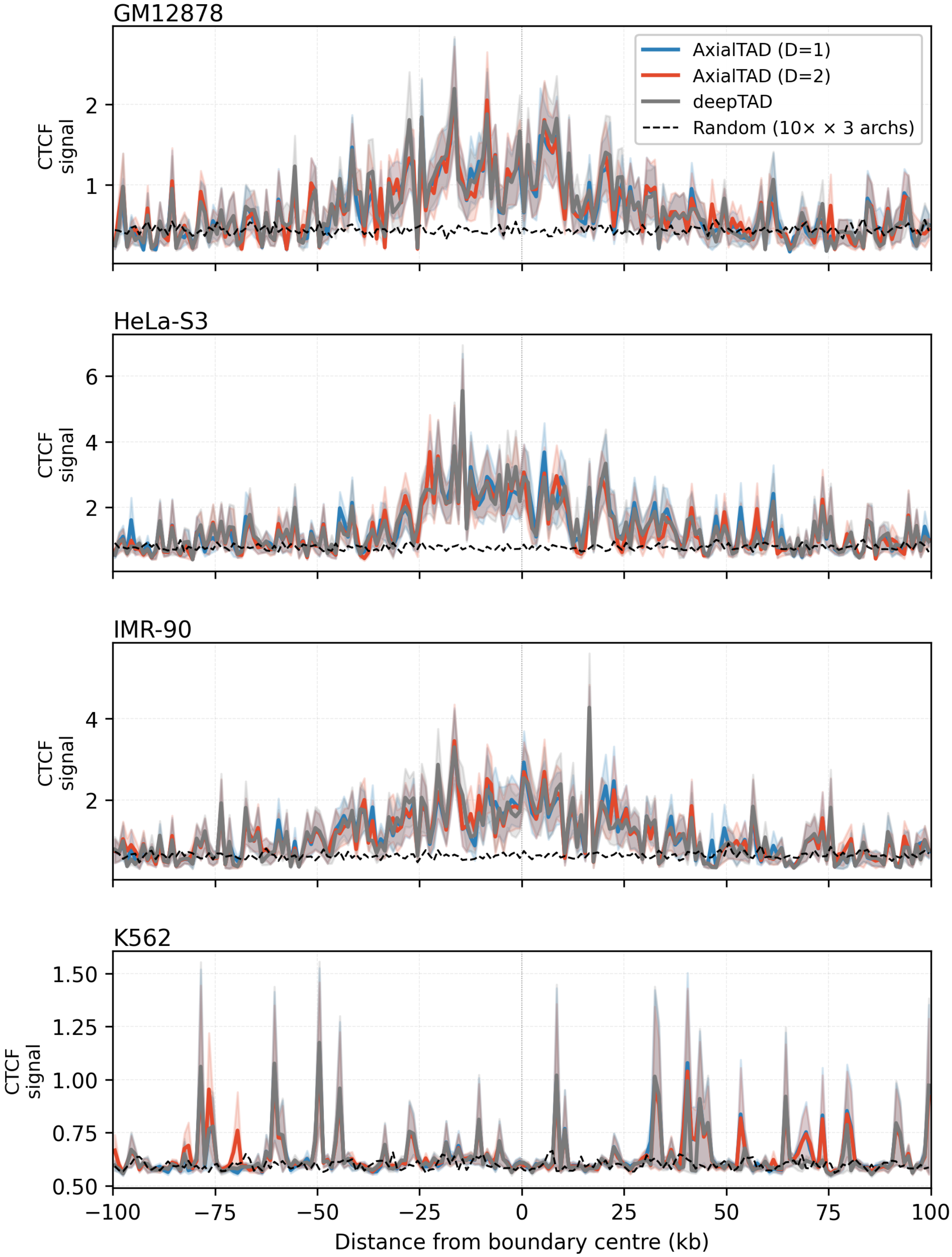

Figure S2: CTCF ChIP-seq signal profiles around predicted boundaries.

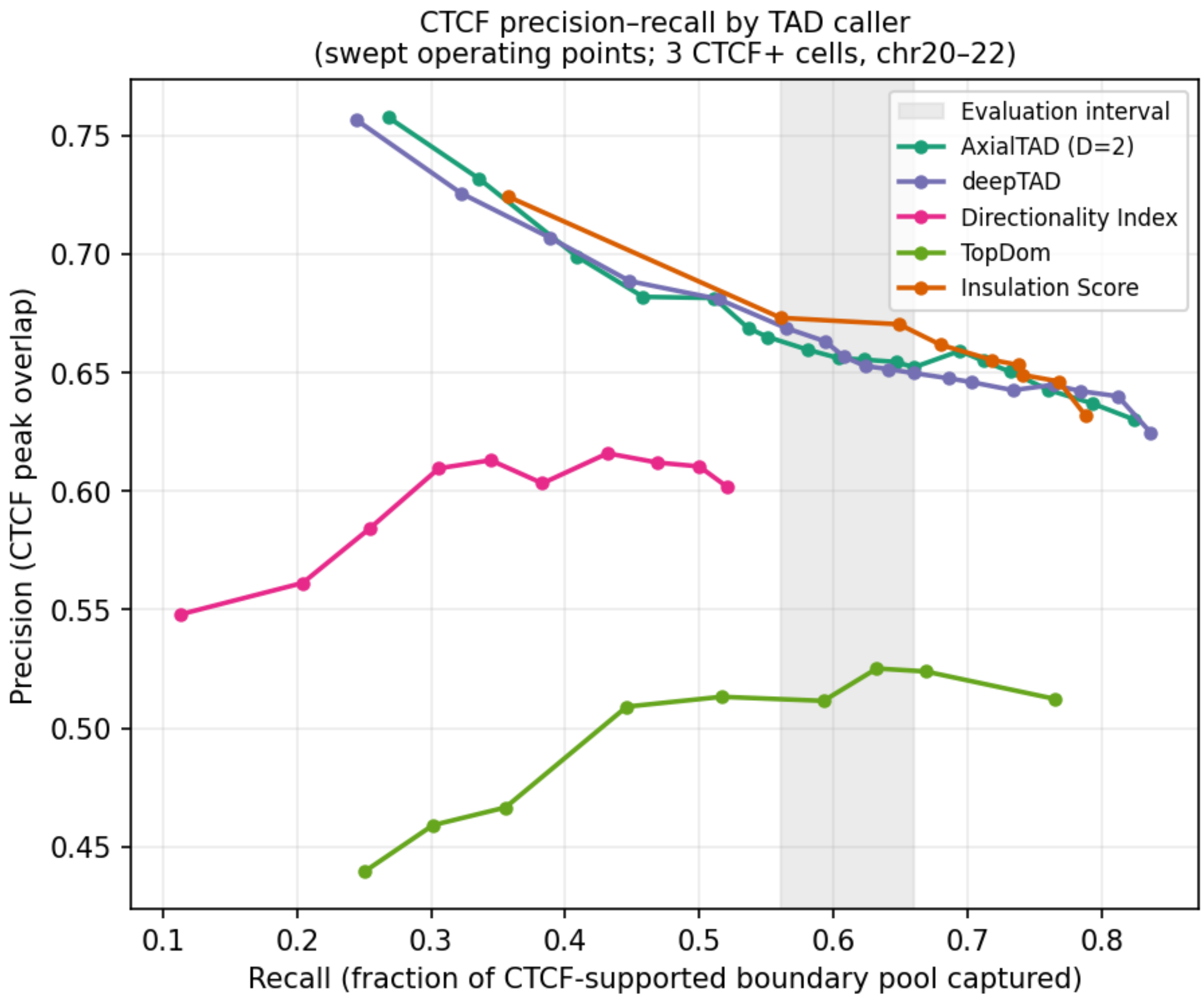

Figure S3: Precision–recall curves for CTCF support across methods, pooled over GM12878, HeLa-S3, and IMR-90. The shaded region indicates the fixed recall interval 0.5607–0.6600 used to calculate normalized partial AUPRC (Table 6). DI did not reach this interval and was therefore excluded from the interval-based summary.

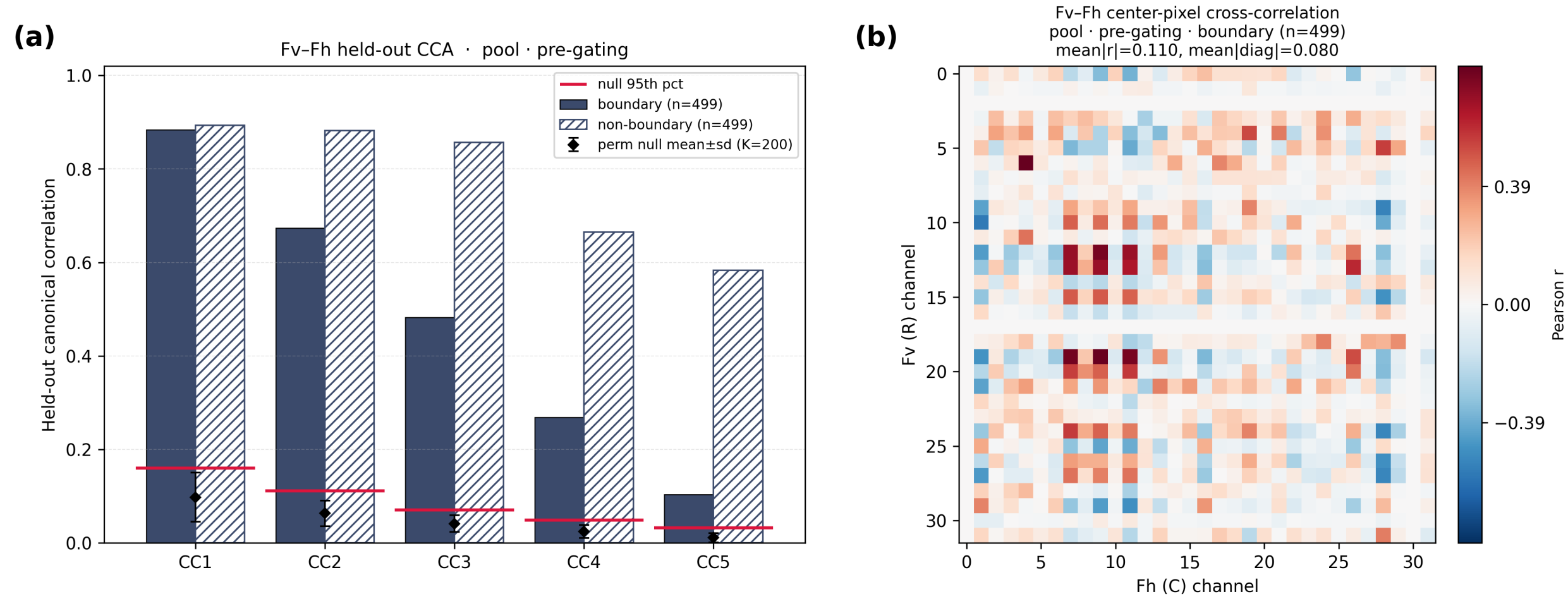

Figure S4: Representational overlap between the vertical (Fv) and horizontal (Fh) axis branches of the ACM: held-out canonical correlations against a permutation null (a) and channel-wise cross-correlation (b), pooled across the four cell lines
